# DNA binding by polycomb-group proteins: searching for the link to CpG islands

**DOI:** 10.1093/nar/gkac290

**Published:** 2022-04-30

**Authors:** Brady M Owen, Chen Davidovich

**Affiliations:** Department of Biochemistry and Molecular Biology, Biomedicine Discovery Institute, Faculty of Medicine, Nursing and Health Sciences, Monash University, Clayton, VIC, Australia; Department of Biochemistry and Molecular Biology, Biomedicine Discovery Institute, Faculty of Medicine, Nursing and Health Sciences, Monash University, Clayton, VIC, Australia; EMBL-Australia, Clayton, VIC, Australia

## Abstract

Polycomb group proteins predominantly exist in polycomb repressive complexes (PRCs) that cooperate to maintain the repressed state of thousands of cell-type-specific genes. Targeting PRCs to the correct sites in chromatin is essential for their function. However, the mechanisms by which PRCs are recruited to their target genes in mammals are multifactorial and complex. Here we review DNA binding by polycomb group proteins. There is strong evidence that the DNA-binding subunits of PRCs and their DNA-binding activities are required for chromatin binding and CpG targeting in cells. In vitro, CpG-specific binding was observed for truncated proteins externally to the context of their PRCs. Yet, the mere DNA sequence cannot fully explain the subset of CpG islands that are targeted by PRCs in any given cell type. At this time we find very little structural and biophysical evidence to support a model where sequence-specific DNA-binding activity is required or sufficient for the targeting of CpG-dinucleotide sequences by polycomb group proteins while they are within the context of their respective PRCs, either PRC1 or PRC2. We discuss the current knowledge and open questions on how the DNA-binding activities of polycomb group proteins facilitate the targeting of PRCs to chromatin.

## INTRODUCTION

Multicellular organisms generate hundreds of different cell identities from a single genome by activating and silencing genes in a coordinated manner. New transcription programs must then be maintained through countless cell divisions. Polycomb group (PcG) proteins predominantly exist in two key groups of complexes that are essential for the maintenance of the repressed state of cell-type-specific genes. Polycomb repressive complex 1 (PRC1) is a group of complexes that collectively maintains gene silencing through monoubiquitylation of lysine 119 of histone H2A (H2AK119Ub) and by ubiquitin independent mechanisms (1,2). PRC2 mono-, di- and trimethylates histone H3 on lysine 27 (H3K27me1/2/3) with the H3K27me3 mark being associated with gene repression (2,3).

PcG proteins are essential for the maintenance of cell identity. They are observed from plants to mammals and are highly conserved in all metazoans (2). Abnormal segmentation and aberrant repression of Hox genes observed in flies carrying a mutation in the gene coding for the PRC1 subunit Polycomb (Pc), led to its identification as a repressor (4). Knockouts of PRC1 or PRC2 core components results in embryonic lethality indicating these complexes are indispensable in mammalian development (5–9). Dysregulation of PRC1 and PRC2 in adult tissues has been implicated in multiple human cancers (10–14).

**Figure 1.**
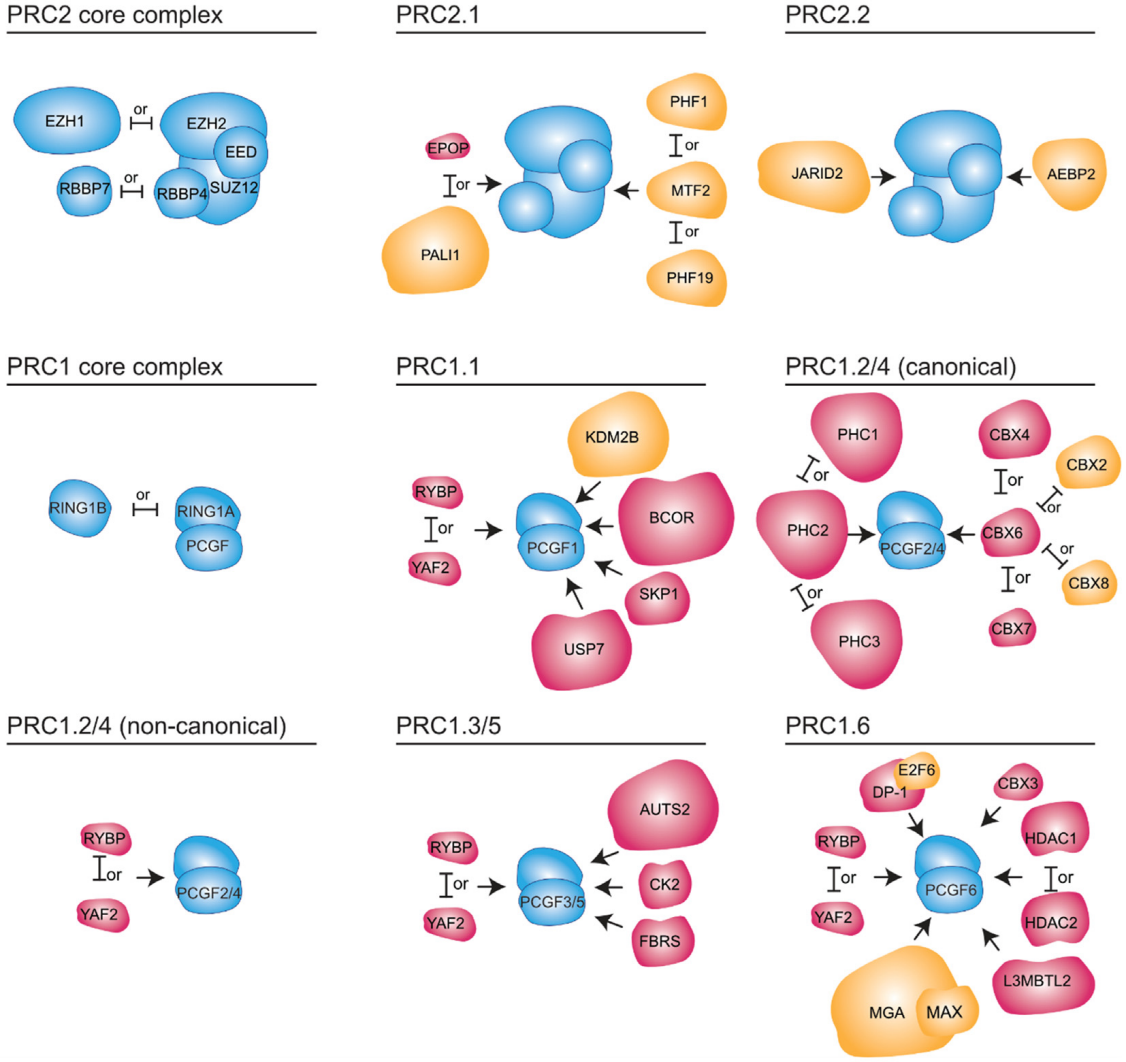
Components of PRC1 and PRC2 complexes in mammals. PcG proteins form two predominant families of complexes; PRC1 and PRC2. Complexes within each family share common core proteins (shown in blue) and interact with accessory subunits (in magenta or yellow) which modulate their enzymatic activity and are required for chromatin binding and correct targeting. Known DNA-binding accessory subunits which may contribute to sequence selective recruitment of PcG proteins are highlighted in yellow. Arrows indicate binding to the core complex, but not necessarily the sites of protein-protein interactions since these are not always known. Mutually exclusive interactions are indicated by ‘or’.

The PRC1 core complex consists of RING1A or RING1B (also termed RING1 and RING2, respectively) in complex with one of the six PCGF subunits. The PCGF protein determines the accessory proteins which bind to PRC1 in vivo and consequently the function of the complex (15,16) (Figure 1). Accordingly, PRC1 complexes are named PRC1.1 to PRC1.6, based on the PCGF protein they associate with (15,16). PRC1.2 or PRC1.4 complexes contain RING1A/B, PCGF2 or PCGF4 and a CBX protein. PRC1.2 and PRC1.4 are sometimes called canonical PRC1 (cPRC1) as the CBX proteins they contain is orthologous to Pc, the first PcG protein identified in Drosophila. Other PRC1 complexes are referred to as non-canonical (ncPRC1) (15–17). PRC1.3 and PRC1.5 complexes are also functionally homologous to each other while PRC1.1 and PRC1.6 form distinct types of ncPRC1 complexes (15,16).

The PRC2 core complex consists of the histone methyltransferase EZH1/2 with EED, SUZ12 and RBBP4/7 (Figure 1). Like in PRC1, the PRC2 core interacts with differentially expressed accessory subunits which influence the function of the complex and are required for its recruitment to chromatin (18,19). The accessory subunits define two types of holo-PRC2 complexes: PRC2.1 and PRC2.2 (16). PRC2.1 consists of one of the polycomb-like (PCL) proteins—PHF1, MTF2 or PHF19—and may include EPOP or PALI1/2. PRC2.2 includes the JARID2 and AEBP2 accessory subunits. The binding of AEBP2 and the PCL proteins to the PRC2 core complex is mutually exclusive (16,20,21).

PRC1 and PRC2 cooperate to repress thousands of shared target genes in a lineage-specific manner. In this review, PcG target genes refers to genes that are marked by both H2AK119ub and H3K27me3, and are transcriptionally silent. PcG target genes may be associated with Polycomb bodies, which are large foci of PcG proteins that can be visualised under a microscope (22). The mechanisms for the recruitment of these complexes to their target genes remain incompletely understood and have been the topic of several recent reviews (3,23–25). Briefly, recognition of histone modifications, DNA and RNA regulate enzyme activity and recruitment to target genes. Notably, PcG proteins bind their own enzymatic products creating positive feedback loops. H2AK119ub is deposited by ncPRC1 and bound by JARID2, which may lead to the recruitment of PRC2 to PRC1 sites (26). PRC2 is allosterically stimulated by its own enzymatic product, H3K27me3, via binding to the PRC2 subunit EED (27,28). CBX proteins also bind to the H3K27me3 mark, leading to the recruitment of cPRC1 to PRC2 bound loci (29).

Up until a decade ago, the contribution of sequence-specific DNA binding by mammalian PRCs was largely dismissed as a contributing factor for their recruitment, since the core PRC1 and PRC2 components lack known DNA-binding domains (30). More recently, it emerges that PRC2 accessory subunits and ncPRC1 bind DNA, though the molecular mechanism and binding specificity are still under investigation (31–38). Sequence selective DNA binding by sub-stoichiometric members of PcG complexes provides a potential mechanistic link between PcG proteins and their target genes. In this review, we discuss developments in the role of DNA binding in the targeting of PcG proteins.

## POLYCOMB RESPONSE ELEMENTS AND THE RECRUITMENT OF PRCs

In Drosophila, DNA sequences known as Polycomb response elements (PREs) contain clusters of transcription factor binding sites. PREs are essential for PcG protein recruitment and both the establishment and maintenance of H3K27me3 in Drosophila (39–42). Pho (Pleiohomeotic) and Phol (Pho-like) were the first proteins shown to bind in a sequence-specific manner to PREs and this leads to the recruitment of PcG proteins (29,43–46). Subsequently, several more transcription factors or their binding motifs were found enriched in PREs and these transcription factors were also proposed to recruit PcG proteins. These transcription factors include SPSS (Sp1 factor for pairing-sensitive silencing), GAF, (GAGA factor), Psq (Pipsqueak), Dsp1 (Dorsal switch protein 1), Grh (Grainy head), Zeste and Combgap (47–49). PcG-mediated gene repression in Drosophila may be explained by the hierarchical model of recruitment (29,50). In this model, the DNA-binding factors Pho and Phol lead to the recruitment of PRC2, via interaction with the EZH1/2 orthologue E(z), which deposits H3K27me3 (29). The H3K27me3 mark is then bound by the chromodomain of Pc (50), the Drosophila homologue of the mammalian CBX proteins of PRC1, leading to gene silencing.

Pho, and to a lesser extent Phol, also interacts with Sfmbt (Scm-like with four MBT domain-containing protein 1) to form the PhoRC complex, a PcG complex distinct from PRC1 and PRC2 (51). Pho and Phol bind at many sites in the genome, including active genes, whereas PhoRC has a narrower distribution that overlaps with PcG target genes and PRC1 (52). PhoRC recruitment to PREs was shown to depend on an interaction between Sfmbt and the PRC1 component Scm (46,52). This observation challenges the hierarchical recruitment model to PREs, where Pho was thought to drive the recruitment of PRC1 and PRC2 to PREs. Instead, this evidence pointed to cooperativity between PRCs and possibly other transcription factors to achieve target specificity at repressed PREs (52).

### Transcription factor recruitment in mammals

While the model of Pho-mediated recruitment to PREs explains PcG binding to a subset of target genes in Drosophila, several lines of evidence, discussed below, suggest this is not the primary mechanism of targeting in mammals.

#### Many PRE-binding proteins lack mammalian orthologues

Pho has a mammalian orthologue named YY1 (reviewed in 53). YY1 binding sites are found throughout the genome and upon its discovery, it was believed to be a missing link to explain the recruitment of PcG proteins to chromatin in mammals. Subsequent work has shown that YY1 has PcG-independent activities (54) and does not colocalise with PRC1 or PRC2 but instead overlaps with H3K4me3 (55). Most other PRE-binding transcription factors either lack mammalian orthologues or their orthologues do not contribute to PcG-mediated repression (53).

#### PRC2 can be recruited by the H2AK119Ub mark deposited by ncPRC1

ncPRC1 can be recruited by PRC2 in mammals, but this interaction is not unidirectional (56). The PRC2.2 accessory subunit JARID2 binds to H2AK119Ub, which may cause the recruitment of PRC2 to some genes marked by PRC1 (26,56). Loss of H2AK119Ub leads to a reduction in H3K27me3 at PcG target sites, highlighting the importance of this interaction (57,58).

#### ncPRC1 localises to PcG target genes independently of H3K27me3 since they lack a CBX protein

In mammals, there are six PRC1 complexes defined by the six different PCGF proteins (15,16). Only PCGF2 and PCGF4 containing cPRC1 complexes associate with a CBX protein and hence can bind H3K27me3 (15,16). CBX-lacking ncPRC1 complexes deposit most of the H2AK119ub in the nucleus (38,56,57,59). Consequently, depletion of SUZ12, which eliminates H3K27me3, has a relatively small effect on H2AK119Ub levels (60). Hence, mammalian cells require H3K27me3-independent mechanisms to recruit ncPRC1 complexes to PcG target genes.

### CpG islands as PREs in mammals

So if not primarily sequence-specific transcription factors, what determines where PcG proteins bind chromatin in mammals? This question may be answered by looking at where PcG proteins deposit their repressive marks in the mammalian genome. Almost all PcG target genes overlap with CpG islands (61). CpG islands are extended regions of DNA with high CpG content that occur despite the global depletion of the CpG dinucleotide in the mammalian genome (62). The C5 position of cytosine lies in the major groove of double-stranded DNA making it accessible to DNA methyltransferases. DNA methyltransferases generate 5-methylcytosine which can undergo spontaneous deamination to form thymine (reviewed in 63). Since DNA methylation occurs predominantly at CpG dinucleotides, the outcome is a high frequency of C to T transitions at CpG sites so this dinucleotide occurs at low frequency throughout the genome (62). CpG islands are believed to resist CpG mutation since they are largely unmethylated in the germline (64–68). Approximately 70% of promoters occur in the vicinity of CpG islands (69). Many orphan CpG islands that—by definition—are located away from annotated genomic elements were proposed to function either as lineage-specific promoters (70) or enhancers (71). This highlights the significance of CpG islands as regulatory elements.

Artificial integration of DNA with properties typical of non-methylated CpG islands and lacking active transcription marks into the mammalian genome in cells is sufficient for PcG protein recruitment (55,72–74). However, only a small proportion of total CpG islands are marked by H3K27me3 in embryonic stem cells (ESCs) and many instead carry the H3K4me3 mark which is associated with active genes or genes that are poised for activation (61,64,75). A group of CpG island promoters in ESCs are marked with both H3K27me3 and H3K4me3 and referred to as bivalent promotors (61,64,66,75,76). Identification of signatures that define PcG target CpG islands from non-targets in each cell type is a critical area of ongoing research. Although sequence-selective transcription factors are not predominant direct drivers of PRC2 localisation to CpG islands, signals for PcG recruitment are intrinsic to the DNA sequence.

## DNA BINDING BY PRC2

**Figure 2.**
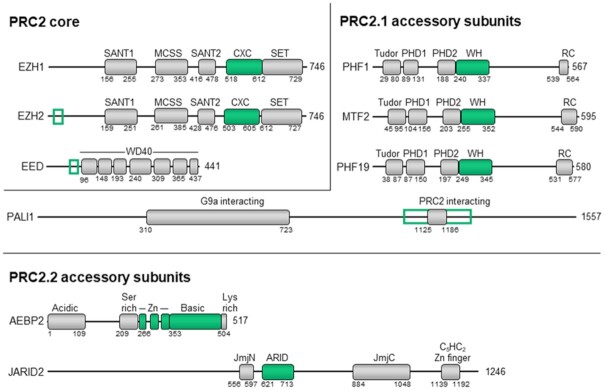
Proposed DNA-binding regions of PRC2 proteins. PRC2 subunits are presented only if DNA-interacting regions were previously identified in them. DNA-binding domains are shown in green. DNA-binding regions outside of defined domains in EZH2 and EED are outlined in green.

PRC2 deposits H3K27me1/2 throughout the genome but high levels of H3K27me3 and detectable binding of PRC2 by ChIP-seq are largely confined to PcG target CpG islands (61,77,78). PRC2 core subunits lack known DNA-binding domains (Figure 2) but DNA binding is believed to be mediated by sub-stoichiometric accessory subunits which have been studied in detail and are the topic of subsequent sections (2,79).

Since both core and accessory subunits are known to bind DNA, studying their contribution to PRC2 recruitment independently has proven challenging. SUZ12 separation-of-function mutants have made this possible. The SUZ12 VEFS domain (SUZ12^VEFS^) assembles with EZH2 and EED and establishes global H3K27me3 levels but this mark is not localised to PcG target genes (80). This SUZ12 mutant cannot bind the DNA-binding accessory subunits of PRC2, indicating that the catalytical core of PRC2 is by itself insufficient to direct PRC2 to target genes. Contrarily, SUZ12 lacking the VEFS domain (SUZ12^ΔVEFS^) cannot interact with EZH2 and, therefore, cannot form a catalytically active PRC2. Yet SUZ12^ΔVEFS^ does interact with accessory subunits and is recruited to PcG target genes (80). This demonstrates the important role of accessory subunits in PRC2 localisation.

Recently, SUZ12 was engineered with mutations that prevented the interaction of PRC2 with either the PCL proteins and EPOP (PRC2.1 accessory subunits), or AEBP2 and JARID2 (PRC2.2 accessory subunits) (33). This provided insights into the relative contribution of each type of subcomplex to PRC2 recruitment. Either PRC2.1 or PRC2.2 is sufficient for the maintenance of pluripotency in human induced pluripotent stem cells but a loss of both families of accessory subunits causes spontaneous differentiation (33). *Phf1*, *Mtf2* and *Phf19* triple knockouts or *Aebp2* and *Jarid2* double knockouts did not lead to a complete loss of H3K27me3 from PcG target genes in mouse embryonic stem cells (19). This indicates that each of the holo-PRC2 complexes are at least partially capable of depositing the H3K27me3 mark to CpG islands. However, the loss of PRC2.1 had a greater impact at CpG islands, showing these complexes are not redundant. DNA-binding functions have been attributed to both PRC2.1 and PRC2.2 accessory subunits (2,79) but the target DNA sequences differ, as discussed below.

While direct interactions between DNA to the catalytic core of PRC2 are insufficient for chromatin targeting, they do take place and are required for normal histone methyltransferase (HMTase) activity. Direct interactions between DNA and the PRC2 core have been observed recently and appear to be independent of DNA sequence (81–84). In one study, recombinant PRC2 was shown to bind a 48 bp long CG repeat DNA with a K_d_ of 32 nM, and the affinity was insensitive to 5′-cytosine methylation (83). Evidence for DNA binding by PcG proteins is summarised in Table 1 and information from quantitative studies where naked DNA is used as the probe are summarised in Table 2. In agreement with these observations, PRC2 HMTase activity is also unaffected by DNA methylation in vitro (85). In another study, the affinity of the PRC2 core complex for non-repetitive DNA sequences was much weaker and this prevented comparison between the affinity for AT- and GC-rich sequences (Table 2, compare rows 1, 2, 6 and 7) (86). At this stage, no sequence has been identified which is selectively bound by the PRC2 core complex across multiple studies from independent laboratories.

**Table 1.** DNA-binding by polycomb-group proteins: a quick guide to the biophysical evidence. Included in the table are only proteins that were shown to bind DNA. The table provides information on the protein construct that was assayed and the binding assays used for confirming the DNA-binding activity and specificity, where applicable. Information about the identified DNA motif is indicated only where probes were designed to test for binding specificity for the sequence, nucleotide composition or the base modifications indicated under ‘Motif’. In cases where ‘n.a.’ is indicated under ‘Motifs’, evidence could not support or was not intended to support binding specificity. In cases where ‘n.d.’ is indicated under ‘Motif’, specificity was not determined although experiments were designed to identify it (commonly, these would include size-matched probers that were subjected to specific perturbations required to test for a hypothesized motif; see Table 2 for a summary of probes used in various studies). Importantly, although ‘n.d.’ under ‘Motif’ indicates that specificity was not determined under the assayed conditions, it should not necessarily be interpreted as nonspecific binding. Unless otherwise indicated, nucleotide sequences and base modifications are referred to as DNA bases within the context of double-stranded DNA. All proteins are of mammalian species unless otherwise indicated. Method abbreviations: electrophoretic mobility shift assay (EMSA), microscale thermophoresis assays (MST), isothermal titration calorimetry (ITC), fluorescence anisotropy (FA), filter binding assay (FBA), systematic evolution of ligands by exponential enrichment (SELEX) and cyclic amplification and selection of targets (CASTing)

Protein		DNA-binding activity			DNA-binding specificity				Comments
Relevant complex	Assayed subunit	Was full-length protein assayed? [Y/N]	Was protein assayed within its PRC? [Y/N]	Method(s) used for detecting DNA-binding activity	Were quantitative binding assays done? [Y/N]	Were probes designed to test for binding specificity? [Y/N]	Motif	Method(s) used for assaying DNA-binding specificity	
PRC2	PRC2 (EZH2, EED, SUZ12 and RBBP4)	Y	Y	EMSA (83), MST (34)	Y	Y	n.d.	EMSA (83), MST (34)	
PRC2.2	AEBP2	Y	Y	EMSA and FA (83)	Y	Y	Methylated CpG DNA is preferred over unmethylated CpG DNA.	EMSA (83,112)	
PRC2.2	AEBP2	N	N	EMSA (96)	N	Y	CTT[N_18-26_]GCC motif concluded.	EMSA (96)	
PRC2.2	JARID2	Y	Y	EMSA (83)	Y	N	n.a.		
PRC2.2	JARID2	Y	N	SELEX (103)	N	N	Slight preference for CG-rich sequences.	SELEX (103)	
PRC2.2	JARID2	N*	N	EMSA (103)	N	N	n.a.		The JmjN, ARID, JmjC and the C5HC2 Zn finger domains are collectively required for DNA binding. (103)
PRC2.2	JARID2	N	N	EMSA (114)	N	N	5′-(T)_4–6_, TATT, or TAAT motifs	CASTing (114)	
PRC2.1	PHF1	Y	Y	EMSA and MST (34)	Y	Y	n.d.	MST (34)	
PRC2.1	PHF1	N	N	ITCand EMSA (31), FA (34), NMR (208)	Y	Y	Two CpG dinucleotides are required but not sufficient for binding.	ITC (31)	
PRC2.1	MTF2	Y	Y	EMSA (32)	Y	Y	G and C bases are required for binding.	EMSA (32)	
PRC2.1	MTF2	Y	?*	DNA pulldown from mouse ESC extract (123)	N	Y	Preferred binding to unmethylated DNA and helical-shape-selectivity concluded.	DNA pulldown from mouse ESC extract (123)	*Immunoblotting for selected subunits carried out.
PRC2.1	MTF2	Y*	N	EMSA (32)	Y	N	n.a.		*Amino acids 1–539 were assayed, which is nearly the full-length protein.
PRC2.1	MTF2	N	N	ITC and EMSA (31)	Y	Y	Two CpG dinucleotides are required but not sufficient for binding.	ITC (31)	
PRC2.1	PHF19	Y	Y	EMSA (33)	Y	Y	Enhanced affinity* to GC-rich sequences.	EMSA (33)	*2- to 4-fold *K_d_* comparing GC-low sequences.
PRC2.1	Pcl (fly)	N	N	FA (34)	Y	Y	n.d.	FA (34)	
PRC2.1	PALI1	N	Y	EMSA (86)	Y	Y	n.d.	EMSA (86)	
PRC2.1	PALI1	N	N	EMSA (86)	Y	N	n.a.		
PRC1	Pc, Ph, Psc and dRing1 (fly)	Y	Y (cPRC1)	FBA (160)	Y	N	n.a.		
PRC1	Psc (fly)	Y	N	FBA (160)	Y	N	n.a.		
PRC1.1	KDM2B	N	N	EMSA (35)	N	N	Non-methylated CpG is preferably bound compared to methylated CpG DNA.	EMSA (35)	
PRC1.1	KDM2B	N	N	EMSA (151)	N	N	n.a.		
PRC1.1	KDM2B	N	N	ITC (155)	Y	Y	Non-methylated CpG is preferentially bound.	ITC (155)	
PRC1.2/4	CBX2	N*	N	EMSA (162)	Y	N	n.a.		* The AT-hook domain identified as sufficient for binding to DNA.
PRC1.2/4	CBX8	N*	N	EMSA (163)	N	N	n.a.		* The chromodomain identified as sufficient for binding to DNA.
PRC1.4	RING1B- PCGF4	N	N	FA (159)	Y	Y	n.d.	FA (159)	
PRC1.6	E2F6-DP2 heterodimer	Y	N	Gel shift (178)	N	Y	DNA from a selected target gene.	Gel shift (178)	
PRC1.6	MAX-MGA	N*	N	Gel shift (172)	N	N	n.a.		*heterodimers including MGA truncations and the full-length MAX were assayed.
PhoRC and possibly other PRCs	Pho (fly)	Y	Y (cPRC1)	EMSA (46)	N	N	n.a.		
PhoRC and possibly other PRCs	Pho (fly)	Y	N	EMSA (46)	N	Y	13-mer motif* derived from PREs is required and sufficient for binding.	EMSA (46)	*The motif identified earlier from PRE sequences (45)

**Table 2.** Summary of quantitative studies of polycomb-group protein binding to naked DNA. For each protein, records are in descending order of *K_d_* values. All DNA is double-stranded unless otherwise indicated. ‘-’ indicates that no binding was detected or that the affinity was too weak to quantify. ‘mC’ denotes 5′-methylcytosine. Abbreviations: Electrophoretic mobility shift assay (EMSA), fluorescence anisotropy (FA), filter binding assay (FBA) isothermal titration calorimetry (ITC), microscale thermophoresis (MST). ^a^ DNA hairpins: the sequence of the double-stranded regions is given, and the underlined sequence is the hairpin loop. The length, CpG number and GC percentage are given for the double-stranded region only. ^b^ The sequence was not given. ^c^ These were titration experiments where the probe concentration was close to or above the *K_d_* concentration. ^d^ May have been PHF1^28-87^. ^e^ Values in brackets represent the 95% confidence interval. ^f^ Hemi-methylated. ^g^ phosphorylated in the serine-rich region

	Protein construct	Substrate	*K_d_* (nM)	Length	Number of CpGs	GC (%)	Method	Reference
	PRC2							
1		(mCpG)24	31 ± 5	48	24	100	EMSA	(83)
2		(CpG)24	32 ± 3	48	24	100	EMSA	(83)
3		TCCCTCTCTC**CG**CAGT**CGCG**G**CG**CAGT**CG**C	64 ± 4	30	5	73	EMSA	(34)
4		CATAAT**CG**TTTGCTGAATCTGAATGGTTTG	150 ± 11	30	1	37	MST	(34)
5		TCCCTCTCTC**CG**CAGT**CGCG**G**CG**CAGT**CG**C	218 ± 22	30	5	73	MST	(34)
6		GG**CG**CCCTGCCC**CG**CCT**CG**CTCTGGCAGAGTGGGGAGCCAGC**CG**GCGCTA^a^	>4000	46	4	80	FA	(86)
7		AATATTTCATTTTATTCTATCTCAATGAGACAAAAGATTGATTAATGCTA^a^	>4000	46	0	20	FA	(86)
	PRC2.2							
8	PRC2-AEBP2	(m**CG**)_24_	11 ± 6	48	24	100	EMSA	(83)
9	PRC2-AEBP2	(**CG**)_30_	15 ± 2	60	30	100	FA	(83)
10	PRC2-AEBP2	60mer^b^	55 ± 9	60			EMSA	(83)
11	PRC2-AEBP2	AATGm**CG**TCCTm**CG**GGTTm**CG**TCCCCAGCm**CGmCG**TCTAm**CGmCG**CCTCm**CG**TCCT	180 ± 40	48	8	69	EMSA	(112)
12	PRC2-AEBP2	(**CG**)_24_	562 ± 47	48	24	100	EMSA	(83)
13	PRC2-AEBP2	(TA)_30_	725 ± 50	60	0	0	FA	(83)
14	PRC2-AEBP2	AATG**CG**TCCT**CG**GGTT**CG**TCCCCAGC**CGCG**TCTA**CGCG**CCTC**CG**TCCT	5,900 ± 600	48	8	69	EMSA	(112)
15	PRC2-AEBP2	GGGCGGCCGCCC	>2000	12	2	100	EMSA	(33)
16	PRC2-AEBP2	ACGCGCGCCGGGAAATTGAAGCGG	>2000	24	5	67	EMSA	(33)
17	PRC2-AEBP2	TCCGCCCGCTCGACGCGCGCCGGGAAATTGAAGCGG	>2000	36	8	72	EMSA	(33)
18	PRC2-AEBP2	GCGCTAGGAGCATCCGCCCGCTCGACGCGCGCCGGGAAATTGAAGCGG	>2000	48	9	71	EMSA	(33)
19	PRC2-AEBP2	TATATAATTATATTATATTAAAAT	>2000	24	0	0	EMSA	(33)
20	PRC2-AEBP2	TCCGCCCGCTCGACGCGCGCCGGGAAATTGAAGCGG	>2000	36	8	72	EMSA	(33)
21	PRC2-AEBP2	ATATTTTATATATATATAAATTAATATATAATTATATTATATTAAAAT	>2000	48	0	0	EMSA	(33)
22	PRC2-AEBP2-JARID2	60mer^b^	46 ± 11	60			EMSA	(83)
	PRC2.1							
23	PRC2-PHF1	TCCCTCTCTC**CG**CAGT**CGCG**G**CG**CAGT**CG**C^c^	30 ± 3	30	5	73	EMSA	(34)
24	PRC2-PHF1_(515–567)_	TCCCTCTCTC**CG**CAGT**CGCG**G**CG**CAGT**CG**C^c^	49 ± 4	30	5	73	EMSA	(34)
25	PRC2-PHF1	CATAAT**CG**TTTGCTGAATCTGAATGGTTTG	117 ± 5	30	1	37	MST	(34)
26	PRC2-PHF1	TCCCTCTCTC**CG**CAGT**CGCG**G**CG**CAGT**CG**C	142 ± 22	30	5	73	MST	(34)
27	PRC2-PHF1_(515–567)_	TCCCTCTCTC**CG**CAGT**CGCG**G**CG**CAGT**CG**C	252 ± 8	30	5	73	MST	(34)
28	PHF1^165-360^	GGG**CG**TA**CG**CCC	3,900 ± 500	12	2	83	ITC	(31)
29	PHF1^165-360^	GGG**CG**CTAG**CG**CCC	11,300 ± 800	14	2	86	ITC	(31)
30	PHF1^165-360^	GGC**CG**GC**CG**GCC	22,000 ± 4,000	12	2	100	ITC	(31)
31	PHF1^165-363^	TCCCTCTCTC**CG**CAGT**CGCG**G**CG**CAGT**CG**C	29,700 ± 500	14	5	73	FA	(34)
32	PHF1^165-360^	GGG**CG**AT**CG**CCC	31,000 ± 2000	12	2	83	ITC	(31)
33	^d^PHF1^14-87^	10 bp^b^	201	10			NMR	(208)
34	PHF1^165-360^	GGA**CG**AT**CG**TCC	-	12	2	67	ITC	(31)
35	PHF1^165-360^	GGA**CG**TA**CG**TCC	-	12	2	67	ITC	(31)
36	PHF1^165-360^	GGT**CG**AT**CG**ACC	-	12	2	67	ITC	(31)
37	PRC2-MTF2	GTGGTGGTGGTGGTTGAGAAAATAAAACCCAGAG**CG**CTAGGAGCATC**CG**CC**CG**CT**CG**A**CGCGCG**C**CG**GGAAATTGAAG**CG**GGGATATTGACAC**CG**ATTCA	15 ± 0.3	100	10	57	EMSA	(32)
38	MTF2^1-539^	GTGGTGGTGGTGGTTGAGAAAATAAAACCCAGAG**CG**CTAGGAGCATC**CG**CC**CG**CT**CG**A**CGCGCG**C**CG**GGAAATTGAAG**CG**GGGATATTGACAC**CG**ATTCA	190	100	10	57	EMSA	(32)
39	MTF2^180-378^	GGG**CG**GC**CG**CCC	2100 ± 300	12	2	100	ITC	(31)
40	MTF2^180-378^	GGT**CG**GC**CG**ACC	6400 ± 1000	12	2	83	ITC	(31)
41	MTF2^180-378^	GGG**CG**CTAG**CG**CCC	9000 ± 2000	14	2	86	ITC	(31)
42	MTF2^180-378^	GGC**CG**GC**CG**GCC	12 000 ± 1000	12	2	100	ITC	(31)
	MTF2^180-378^	GGG**CG**AT**CG**CCC	22 000 ± 7000	12	2	83	ITC	(31)
43	MTF2^180-378^	GGG**CG**TA**CG**CCC	25 000 ± 4000	12	2	83	ITC	(31)
44	MTF2^180-378^	GGA**CG**GC**CG**TCC	33 000 ± 6000	12	2	83	ITC	(31)
45	MTF2^180-378^	GGA**CG**AT**CG**TCC	-	12	2	67	ITC	(31)
46	MTF2^180-378^	GGA**CG**TA**CG**TCC	-	12	2	67	ITC	(31)
47	MTF2^180-378^	GGT**CG**AT**CG**ACC	-	12	2	67	ITC	(31)
48	PRC2-MTF2	TATATATATTTTATATATATATAAATTAATATATAATTATATTATATTAAAATTAATATTATATTTAAATTATTATATATATATAAATAATTTAATTATA	-	100	0	0	EMSA	(32)
49	PRC2-PHF19	TCCGCCCGCTCGACGCGCGCCGGGAAATTGAAGCGG	10 (6,30) ^e^	36	8	72	EMSA	(33)
50	PRC2-PHF19	ATATTTTATATATATATAAATTAATATATAATTATATTATATTAAAAT	20 (20,30) ^e^	48	0	0	EMSA	(33)
51	PRC2-PHF19	GCGCTAGGAGCATCCGCCCGCTCGACGCGCGCCGGGAAATTGAAGCGG	40 (30,60) ^e^	48	9	71	EMSA	(33)
52	PRC2-PHF19	TATATAAATTAATATATAATTATATT ATATTAAAAT	40 (30,70) ^e^	36	8	72	EMSA	(33)
53	PRC2-PHF19	ACGCGCGCCGGGAAATTGAAGCGG	370 (220, 620) ^e^	24	5	67	EMSA	(33)
54	PRC2-PHF19	TATATAATTATATTATATTAAAAT	1600 (810, 3900) ^e^	24	0	0	EMSA	(33)
55	PRC2-PHF19	GGGCGGCCGCCC	>2000	12	2	100	EMSA	(33)
56	Pcl^491-694^	TAATGGCTG**CG**C**CG**TAAAGC	2900	20	2	55	FA	(34)
57	Pcl^491-694^	CTCTC**CG**CAGT**CGCG**G**CG**CA	3500	20	4	75	FA	(34)
58	Pcl^491-694^	**CG**TG**CG**TAAGAG**CG**AGATAC	4200	20	3	55	FA	(34)
59	Pcl^491-694^	GC**CG**TAAAG**CG**AGAG**CG**ATC	4200	20	3	60	FA	(34)
60	Pcl^491-694^	GT**CG**CCATAACTGT**CG**TT**CG**	4200	20	3	55	FA	(34)
61	Pcl^491-694^	GTTTGCTGAATCTGAATGGT	4200	20	0	40	FA	(34)
62	Pcl^491-694^	CTGT**CG**TT**CG**TAATGGC**CG**T	4400	20	3	55	FA	(34)
63	Pcl^491-694^	**CG**AG**CG**AGAAGGCTAAC**CG**T	5100	20	3	60	FA	(34)
64	Pcl^491-694^	CCTCATAAT**CG**TTTGCTGAA	5100	20	1	40	FA	(34)
65	Pcl^491-694^	A**CGCG**CACCATAATGGCTGC	7300	20	2	60	FA	(34)
66	Pcl^491-694^	TTTAAGTG**CG**ACTGAGATGG	7300	20	1	45	FA	(34)
67	Pcl^491-694^	TAATGGC**CG**TTTTAAGTG**CG**	7600	20	2	45	FA	(34)
68	Pcl^491-694^	ATCTCTCCCTCTCTC**CG**CAG	7700	20	1	60	FA	(34)
69	Pcl^491-694^	ACTGAGATGGCCTCATAATC	8000	20	0	45	FA	(34)
70	Pcl^491-694^	GGCTAAC**CG**TATCTCTCCCT	8700	20	1	55	FA	(34)
71	Pcl^491-694^	CTGCAGCTC**CG**T**CG**CCATAA	9000	20	2	60	FA	(34)
72	Pcl^491-694^	TCCCTCTCTC**CG**CAGT**CGCG**G**CG**CAGT**CG**C	9700 ± 200	30	5	73	FA	(34)
73	Pcl^491-694^	AGATAAGACTA**CGCG**CACCA	9800	20	2	50	FA	(34)
74	Pcl^491-694^	AG**CG**AGATACAGATAAGACT	11400	20	1	40	FA	(34)
75	Pcl^491-694^	GAGAG**CG**ATC**CG**AG**CG**AGAA	13300	20	3	60	FA	(34)
76	PRC2-PALI^1058-1250^	AATATTTCATTTTATTCTATCTCAATGAGACAAAAGATTGATTAATGCTA^a^	73.7 ± 10	46	0	20	EMSA	(86)
77	PRC2-PALI^1058-1250^	GG**CG**CCCTGCCC**CG**CCT**CG**CTCTGGCAGAGTGGGGAGCCAGC**CG**GCGCTA^a^	155 ± 26	46	4	80	EMSA	(86)
	PRC1							
78	Pc-Ph-Psc-dRing1	155 bp^b^	0.13 ± 0.08	155			FBA	(160)
79	Psc	155 bp^b^	0.15 ± 0.08	155			FBA	(160)
	PRC1.1							
80	KDM2B^607–723^	GCCAC**CG**GTGGC	2200 ± 300	12	1	83	ITC	(155)
81	KDM2B^607–723^	GCCAG**CG**CTGGC	1600 ± 400	12	1	83	ITC	(155)
82	KDM2B^607–723^	GCCAA**CG**TTGGC	2500 ± 500	12	1	67	ITC	(155)
83	KDM2B^607–723^	GCCAT**CG**ATGGC	2200 ± 300	12	1	67	ITC	(155)
84	KDM2B^607–723^	GCCAGTACTGGC	NB	12	0	67	ITC	(155)
85	KDM2B^607–723^	GCCAAAmCGTAACCG^f^	NB	12	0	67	ITC	(155)
	PRC1.4							
86	RING1B^1-116^-PCGF4^1-109^	GTGTTACTAGCT	2800 ± 300	12	0	42	Competition FA	(159)
87	RING1B^1-116^-PCGF4^1-109^	TCAGCTGAACAT	3200 ± 300	12	0	42	FA	(159)
88	RING1B^1-116^-PCGF4^1-109^	GGACCTGATGAC	3200 ± 300	12	0	58	Competition FA	(159)
89	RING1B^1-116^-PCGF4^1-109^	AAGACATA**CG**AG	3700 ± 200	12	1	42	Competition FA	(159)
90	RING1B^1-116^-PCGF4^1-109^	TCAGCTGAACAT	3800 ± 300	12	0	42	Competition FA	(159)
91	RING1B^1-116^-PCGF4^1-109^	TCAGCTGAACAT	4100 ± 300	12	0	42	Competition FA	(159)
91	RING1B^1-116^-PCGF4^1-109^	CTAGCCTAG**CG**A	4300 ± 200	12	1	58	Competition FA	(159)
93	RING1B^1-116^-PCGF4^1-109^	TCAGCTGAAC	6400 ± 900	10	0	50	Competition FA	(159)
94	RING1B^1-116^-PCGF4^1-109^	TCAGCTGA	12000 ± 2000	8	0	50	Competition FA	(159)
95	RING1B^1-116^-PCGF4^1-109^	TCAGCT	75000 ± 44000	6	0	50	Competition FA	(159)
96	CBX2^2-63^	Major satellite	8110 ± 360	130			EMSA	(162)
97	CBX2^60-82^	Major satellite	510 ± 50	130			EMSA	(162)
98	CBX2^2-82^	Major satellite	320 ± 50	130			EMSA	(162)
99	CBX2^2-129^	Major satellite	210 ± 10	130			EMSA	(162)
100	CBX2^2-129 g^	Major satellite	>10000	130			EMSA	(162)

Electrostatic interactions between EED or EZH2 and DNA may explain sequence-independent binding. Several recent structures of PRC2 bound to nucleosomes have shown that DNA makes direct contact with the PRC2 core subunits EZH2 and EED at sites rich in basic residues (81,82,84). At least some of these basic residues, at the interface between the catalytic core of PRC2 to the nucleosomal DNA, are required for histone methyltransferase (84). A structure of PRC2-AEBP2 bound to a dinucleosome shows the complex can simultaneously interact with both a substrate nucleosome, which has the tail of histone H3 positioned in the EZH2 active site, and a regulatory nucleosome that contacts the opposite face of the protein (81). These multiple contact sites are consistent with earlier findings that the HMTase activity is higher on oligonucleosomes than mononucleosomes or histone proteins (87). When PRC2 is bound to the dinucleosome construct, the CXC and SET domains of EZH2 sit close to DNA where it exits the substrate nucleosome. Contacts exist between DNA and a lysine-rich region of the CXC domain spanning residues 561–570 (81). A nucleosome-bound structure of PRC2-AEBP2-JARID2^1-450^ generally agreed with these substrate nucleosome interactions (82). This structure showed an additional positive patch of EZH2 spanning residues 487–513 which contacts DNA and this includes Lys509 and Lys510 (82), which are automethylated (88,89). EZH1 containing PRC2 also contacts DNA through the CXC domain (90). Returning to the context of the dinucleosome construct, contacts with the non-substrate nucleosome are mediated by two positive patches in the SANT binding domain of EZH2: 16–RKRVK-20 and 27–RQLKR-30 (81). A positive surface along EED makes additional contacts with DNA though these contacts varied with the orientation of the non-substrate nucleosome (81). The lysine-rich region of EED 70–KGKWKSKKCK-79 is also hypothesised to interact with the DNA backbone (81). These lysine- and arginine-rich sequences in the core PRC2 complex might provide a rather broad target selectivity, leaving more selective tethering to the accessory subunits or other factors. Yet, at this time, little is known about the selectivity of PRC2 to DNA sequences in the context of nucleosomes.

The two catalytic subunits of PRC2—EZH1 and EZH2—have distinct DNA-binding properties. EZH2 is the most abundant isoform in proliferating cells while EZH1 dominates in differentiated adult tissues (91). EZH2 has higher HMTase activity in vitro and accounts for the majority of the H3K27me3 mark in proliferating cells (91,92). PRC2:EZH1 has a higher affinity for DNA than PRC2:EZH2. This may be due to the loop between the MCSS and SANT2 domains (Figure 2) which is rich in basic residues. In EZH1 the MCSS/SANT2 loop is positioned near DNA (90). The same loop in EZH2 has a large acidic insertion which may prevent these interactions (90). The HMTase activity of PRC2:EZH1 is inhibited by overhanging DNA on nucleosomes or competitor DNA (92). In contrast, the HMTase activity of PRC2:EZH2 is higher on nucleosomes with overhanging DNA compared to the minimal nucleosome core particle (83,92). PRC2:EZH1 has also been reported to compact chromatin independently of its HMTase activity (90,91). This may be mediated by DNA interactions since it is dependent on the basic residues in the MCSS/SANT1 loop (90). Although both PRC2:EZH1 and PRC2:EZH2 bind DNA, evidence of distinct biological roles for this shared function is still emerging. Collectively, this evidence fit with a model were contacts between the catalytical core of PRC2 to nucleosomal DNA are largely dispensable for selective targeting in cells but are required for histone methyltransferase. In the case of PRC2:EZH1, interactions with DNA might possibly also contribute to chromatin compaction.

### PRC2.2 subunits enhance DNA binding with undetermined sequence selectivity

AEBP2 was first identified as a Zinc finger containing transcriptional repressor in mice (93). JARID2 was first identified as a protein necessary for neuronal development (94,95). Both AEBP2 and JARID2 were subsequently shown to interact with PRC2 core proteins and colocalise to PcG target genes (20,96–101). AEBP2 and, to a greater extent, JARID2 stimulate the HMTase activity of PRC2 in vitro and the two proteins can act synergistically for maximal activity (82,87,102). Both AEBP2 and JARID2 contain predicted DNA-binding domains (Figure 2) and have been proposed to recruit PRC2 to PcG target genes through interactions with DNA. JARID2 is necessary for the recruitment of PRC2 to at least a subset of target genes (97,101,103), but the same has not been clearly shown for AEBP2. Further complicating the story, JARID2 binds the H2AK119Ub mark deposited by PRC1, which provides a DNA-sequence independent recruitment mechanism (26). AEBP2 is also reported to interact with the H2AK119Ub modification (82). The relationship between PcG recruitment and the DNA-binding activity of the PRC2.2 complex has been explored extensively since its discovery. However, what DNA sequences are recognised by the PRC2.2 complex, which domains contact the DNA and how these affects the localisation of PRC2 and H3K27me3 are still open questions.

#### DNA binding by the zinc-finger protein AEBP2

AEBP2 enhances PRC2–chromatin binding by interacting with DNA. Longer linker DNA increases the affinity of PRC2-AEBP2 for nucleosomes. Mononucleosomes assembled on a 147 bp Widom 601 sequence—a non-natural sequence that was selected for high octamer stability (104)—with no overhanging DNA were bound by PRC2-AEBP2, with an apparent *K_d_* of 41 000 nM compared to an apparent K*_d_* of 280 nM when the DNA length was increased to 207 bp (83). Strikingly, naked DNA was the preferred ligand, with an apparent *K_d_* of 26 nM (83). A similar trend was observed for dinucleosomes, where PRC2-AEBP2 binding increased as the DNA linker length was increased (83). The histone modifications H3K27me3 and H3K4me3 as well as the histone mutation H3K27M are all known to affect the affinity for histone H3 tail peptides and the HMTase activity of PRC2 (27,105–107). However, these histone modifications had minimal effect on the affinity of PRC2-AEBP2 for nucleosome arrays (83), which further supports DNA as the key driver of PRC2-AEBP2 affinity for chromatin.

Several studies have attempted to identify how AEBP2 contacts DNA using truncations of this protein in the absence of PRC2, but the results have been conflicting. AEBP2 contains three Cys_2_His_2_ zinc fingers (Figure 2), a domain that binds nucleic acids in other proteins (see 108,109 for reviews). DNA-binding activity was observed for the zinc fingers of mouse AEBP2 when they were tested as a truncated protein (AEBP2^223-348^), but not for the full-length AEBP2 (96). For the human protein, neither the full length AEBP2 nor the zinc fingers (AEBP2^258-357^) bound DNA (110).

In the context of PRC2, AEBP2 seems to bind DNA via a basic patch downstream of the Zinc fingers. Both the zinc fingers and basic patch are highly conserved in mammals, fish, and insects (96). Enhancement of nucleosome binding and the catalytic activity of PRC2 by AEBP2 was mapped to a stimulator region in the basic domain, spanning residues 381–404 of AEBP2 (92) (Figure 2). A region rich in positive residues, AEBP2 387-KRRKLKNKRRR-397, was proposed to mediate nucleosome binding. Mutating arginine and lysine residues to alanine in either regions 387–390 or 394–397 reduced the nucleosome-binding affinity and the double mutant had a synergistic effect (92). A subsequent structure of PRC2-AEBP2 showed this motif directly contacts DNA (82). This structure also showed that the first two zinc fingers of AEBP2 interact with the H2AK119Ub modification deposited by PRC1 (82). This may explain why the zinc coordinating His315 and the adjacent Ser316 in the second zinc finger in mouse AEBP2 are dispensable for DNA binding in vitro but required for gene repression in vivo (93).

Compared to the other accessory proteins, there have been relatively few investigations into the sequence selectivity of PRC2-AEBP2 DNA binding. Kim *et al.* (96) performed non-quantitative binding studies using mutants of the T1 sequence that was bound by the mouse AEBP2 zinc fingers. They concluded a CTT and GCC sequence separated by an 18–26 bp linker was preferentially bound by both AEBP2^223-496^ and AEBP2^223-348^ in vitro. This motif was also common at AEBP2 target sites in mouse brain tissue (96). The human PRC2-AEBP2 complex preferentially binds 60 bp CG repeats compared to AT repeats (Table 2, compare rows 9 and 13) (83). However, GC selectivity was not observed when a longer DNA was used: Lambda DNA, with a low GC and CpG content, binds PRC2-AEBP2 with a comparable affinity to the same sequence either in the presence or absence of a centrally inserted 200 bp from the *Zfpm2* CpG island (111).

A fully methylated CpG repeat sequence was bound with approximately 50-fold higher affinity than the equivalent non-methylated sequence (Table 2, compare rows 8 and 12) (83). Methyl-selective DNA binding requires the conserved cysteine and histidine residues in the three C_2_H_2_ zinc finger domains of AEBP2 (83). So, although the C_2_H_2_ zinc fingers of AEBP2 bind DNA extremely weakly, they may contribute to DNA sequence selectivity while AEBP2 is in a holo-PRC2 complex. Preferential binding to methylated CpG dinucleotides in vitro contrasted genome-wide studies, where PRC2 is known to localise at non-methylated CpG islands (61,77,78). Therefore, AEBP2 may contribute to the recruitment of PRC2 to methylated CpG DNA in specific cellular contexts. For instance, H3K27me3 and 5-methylcytosine co-occur at the TERT promoter in cancer cells. Accordingly, PRC2-AEBP2 preferably binds to a methylated CpG DNA sequence from the TERT promoter, compared to the non-methylated counterpart (Table 2, compare rows 11 and 14) (112). Considering all available data, it appears that PRC2-AEBP2 binds DNA through its basic region and possibly the zinc finger domains. However, currently, there is no evidence for the direct involvement of DNA-sequence motifs in the targeting of AEBP2 to non-methylated CpG islands in vivo.

#### JARID2 binds DNA through multiple domains

JARID2 contains two known DNA-binding motifs, an ARID domain and a zinc finger, which instigated the hypothesis that DNA recruitment may contribute to the PRC2 targeting by JARID2. The ARID domain of JARID2 binds DNA in vitro (113,114) and is required for SUZ12 binding to target genes and H3K27me3 deposition in ESCs (98). JARID2 also contains a C_5_HC_2_ zinc finger. Zinc fingers are largely recognised as DNA-binding domains although they may also bind proteins, lipids or RNA (115). A direct role of the JARID2 zinc finger in DNA binding is yet to be shown.

Two studies have attempted to determine the sequence selectivity of JARID2 using unbiased in vitro methods. JARID2 truncations encompassing the JmjN and the ARID domains, either with (JARID2^529-1198^) or without (JARID2^529-798^) the JmjC and the C_4_HC_2_ domains, were used to select for preferred DNA-targeting sequences from a pool of random 30 bp DNA oligomers. Based on these experiments, TATT and TAAT were proposed as optimal sequences, although the protein constructs also bound DNA oligos lacking these AT-rich stretches (114).

In contrast to these findings, a SELEX experiment using the full-length JARID2 showed no consensus binding motif but a slight enrichment of CG rich sequences (103). Notably, the ARID domain alone could not bind these DNA sequences. Instead, JARID2^534-1228^ encompassing the JmjN, ARID, JmjC and the C_5_HC_2_ Zn finger domains was needed for DNA binding (103). In support of CG-rich DNA binding, JARID2 is known to bind the CG-rich sequence spanning −187 to −52 in the mouse Cyclin D1 promoter (116). In vivo, a tandem repeat of CCG and a GA-rich motif were enriched in JARID2 target genes (101), consistent with PRC2 localisation at CpG islands (61). Sequence selectivity is possibly conferred by multiple domains in JARID2, explaining the discrepancy when using these different protein constructs, but this is yet to be shown. Furthermore, there is evidence JARID2 stability and chromatin binding is dependent on the PRC2 core in vivo (98,101,103). Further studies into DNA sequence selectivity of JARID2-bound holo-PRC2 complexes may resolve some of the inconsistencies between studies around the DNA-binding specificity of JARID2.

**Figure 3.**
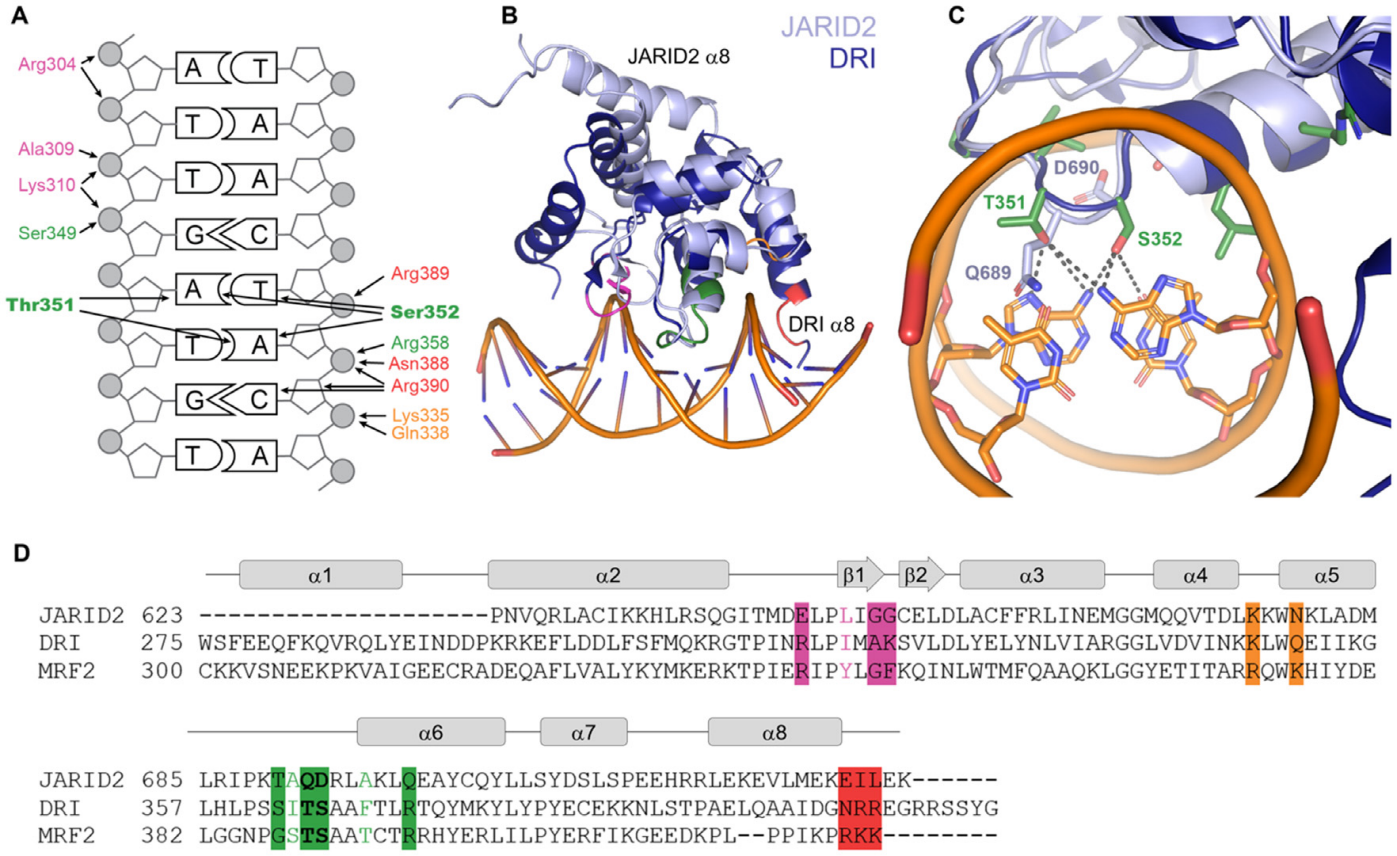
ARID domain mechanism of DNA binding. (**A**) Hydrogen bonds and salt bridges between the ARID domain of DRI and DNA are shown with black arrows. DRI Thr351 and Ser352 are shown in bold and make direct contacts with DNA bases which may confer AT selectivity. Contacts were mapped based on the structure of the ARID domains from DRI in a complex with DNA (PDB ID: 1KQQ (118)). (**B**) The structure of the ARID domain of DRI (dark blue, PDB ID: 1KQQ (118)) with DNA was superimposed over the structure of the ARID domain from JARID2 that was solved without DNA (light blue, PDB ID: 2RQ5 (121)). (**C**) Close contacts between Thr351 and Ser352 (green) of DRI and the DNA bases are indicated in dashed black lines. The equivalent residues in JARID2 are labelled in light blue. (**D**) Sequence alignment of JARID2, DRI and MRF2 ARID domains with DRI DNA contacts from (A) highlighted. In all panels, amino acids in DRI from the loop between H5 and H6 contact bases in the major groove and are labelled in green. Additional interactions are mediated by amino acids in the loop between H2 and H3 (magenta), a pocket between H4 and H5 (orange) and the end of H8 (red).

Experiments using JARID2 truncations suggested an essential role for the ARID domain in DNA binding (103,114). ARID domains were named because those first identified bound AT-rich DNA, although many ARID domain proteins known today show no sequence selectivity (117). Three structures of ARID domains bound to DNA have been solved: Drosophila Dead ringer (DRI) (118), human MRF-2 (119) and *Arabidopsis thaliana* ARID5 (120). AT selective DNA binding is mediated by conserved residues in the loop connecting H5 and H6, which contact DNA bases in the major groove (Figure 3 in green). The interactions with DNA are stabilised by additional contacts with the loop between H2 and H3 (Figure 3A, B, D in magenta) and a pocket formed between helices 4 to 6 (Figure 3A, B, D in orange) (118). An NMR structure of the apo JARID2 ARID domain (JARID2^615-730^) shows a similar architecture to other ARID domain proteins. However, the two residues which contact the AT bases in other ARID domain proteins (e.g. Thr351 and Ser352 in the ARID domain protein DRI) are not evolutionary conserved (Figure 3C, D, in green) (121). If the corresponding residues of JARID2 interact with DNA, despite one of them—JARID2 Asp690—being negatively charged, they may adopt different interactions than seen in the DRI-DNA complex. This may be accompanied by a difference in DNA-sequence-selectivity or no selectivity. Other amino acids which contact DNA in DRI are also poorly conserved in JARID2, but the DNA-binding surface of both proteins has an overall positive charge which may mediate contact with DNA (118,121).

In DRI, the end of helix 8 makes additional contacts with DNA, with the aid of positively charged residues at its C-terminal end (Figure 3A, B, D in red) which is a feature of extended ARID domains (118,119). This is unlikely the case in JARID2, where helix 8 adopts a dramatically different conformation (Figure 3B). ARID domains which lack these interactions bind to DNA with lower affinity and these proteins often contain additional DNA-binding domains (118). In line with this prediction, NMR quantified lower changes in chemical shifts upon the addition of DNA to a JARID2 construct compared to DRI, suggesting weaker DNA binding (121). Collectively, structural and functional data support a role for the ARID domain of JARID2 in DNA binding (103,118,119,121), possibly facilitated by basic residues at its DNA-binding surface, as predicted by homology (Figure 3). Yet, more studies are required to determine if the ARID domain of JARID2 binds DNA in a sequence-specific manner.

Determining the affinity and specificity of JARID2 to DNA in the context of chromatin can be challenging for several reasons. First, dissecting the nucleosome binding from the DNA-binding activities of JARID2 can be difficult since the N-terminal region of JARID2 can bind to H2AK119ub-modified nucleosomes (26). Second, JARID2 is present in the same PRC2 complex as AEBP2. Therefore, separating the DNA-binding activity of JARID2 from that of AEBP2 can be challenging in cells, especially as AEBP2 is required for the efficient incorporation of JARID2 into PRC2 (20). Notably, quantitative binding assays using an intact PRC2-AEBP2-JARID2 complex demonstrated a surprisingly small effect on the affinity to trinucleosomes and naked DNA, relative to the PRC2-AEBP2 complex (83). Finally, like with AEBP2, the apparent sequence selectivity has varied with different protein constructs.

Several studies support a role for the ARID domain (113,114) and possibly also other domains in the large C-terminal region of JARID2 (103) in DNA binding. However, so far there is no consensus on the DNA-binding specificity of JARID2 and no structural data to explain DNA-sequence recognition. Future biophysical and structural studies may reveal the contribution of the different domains in JARID2 to the chromatin and DNA-binding activities of the PRC2.2 complex.

### PRC2.1 as a potential link to non-methylated GC-rich DNA

#### The PCL proteins as a link to CpG islands

PCL proteins have been proposed to link PRC2 to CpG island DNA (31,122,123). The PCL proteins stimulate the HMTase activity of PRC2 in vitro (124) and are required for PRC2 recruitment and high levels of H3K27me3 deposition at PcG target genes in vivo (19,31,122,123,125–129). PCL proteins interact with two components of chromatin which may contribute to their targeting to chromatin: H3K36me2/3 modified histones and DNA. The H3K36me3 mark is proposed to facilitate the recruitment of PRC2 to new target genes during differentiation (125,130,131) or the early DNA-damage response (132), but this mechanism and its functional consequences are not fully understood. PCL proteins increase the affinity of PRC2 for DNA and prolong the average residence time on chromatin (33,34).

**Figure 4.**
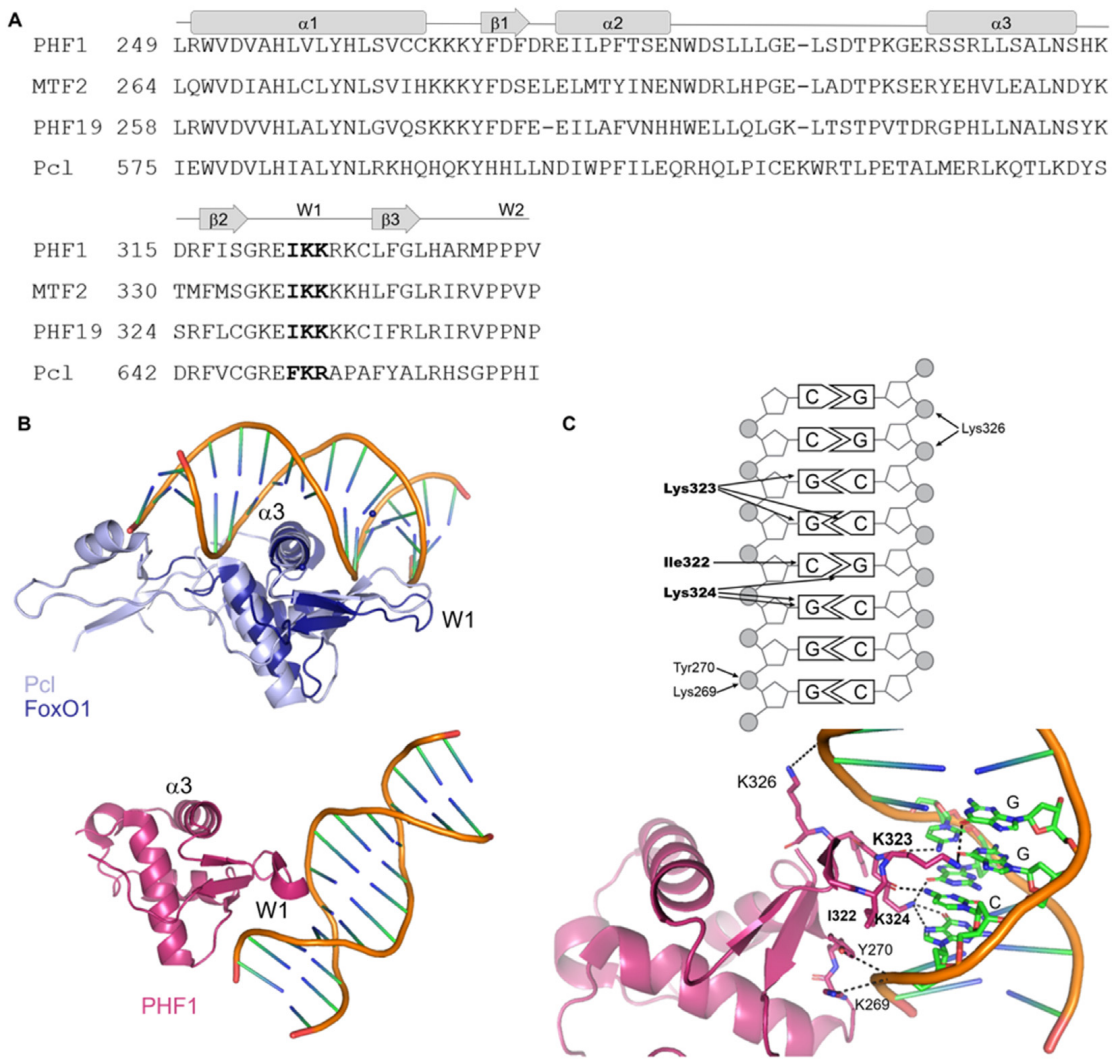
Mechanism of DNA binding by PCL proteins. (**A**) Sequence alignment of WH domains. Amino acids in the conserved Ile-Lys-Lys motif are shown in bold. (**B**) Top structure: A DNA-binding mode of Drosophila Pcl (light blue, PDB ID: 5OQD) proposed by Choi *et al.* (34) based on alignment to the DNA bound structure of the FoxO1 WH domain (dark blue) (PDB ID: 3CO6 (228)). Bottom structure: The DNA-bound structure of PHF1 (magenta), which forms non-canonical interactions with the DNA (PDB ID: 5XFQ (31)). (**C**) Contacts between PHF1 and the CpG dinucleotide. Contacts as reported in Li *et al.* (31) are represented by arrows (top) and dashed lines (bottom). Amino acids which contact the DNA bases, and hence are proposed to contribute to sequence selective binding (31) are in bold.

PCL proteins have a winged-helix (WH) domain that binds DNA though the mechanism is disputed (31,34). WH domains are common in transcription factors and bind DNA by insertion of their α3 helix into the major groove of the DNA (Figure 4A, B). Additional interactions between WH domains and DNA commonly involve their wing 1 (W1) loop contacting the adjacent minor groove, and sometimes a wing 2 (W2) loop (Figure 4A, B). Despite the conserved structure, the amino acid conservation between WH domains is low and, consequently, they vary widely in their DNA sequence selectivity (reviewed in^133^). Li *et al.* (31) solved crystal structures of constructs including the Tudor, PHD1, PHF2 and WH domains from human PHF1 and MTF2 in a complex with a CpG-containing DNA. This structure showed an atypical DNA-binding mode where Lys322 and Lys323 (using the PHF1 numbering, marked in bold black text in Figure 4) on the W1 loop make multiple contacts with the DNA bases of the CGG sequence (Figure 4B, C, in magenta). Lys322 and Lys323 are conserved between all human PCL proteins and the Drosophila Pcl. Mutating them to alanine reduces the affinity of the PCL proteins for DNA (31,32,34). Lys269, Tyr270 and Lys326 further stabilise binding by interacting with the DNA backbone (Figure 4C). The orientation of the DNA precludes any contact with the α3 helix (Figure 4B). A noncanonical binding mode between a WH domain and DNA has been observed previously between the winged helix domain of RFX1 and DNA. However, in this example, the α3 helix of RFX1 still contacts the DNA via the minor groove (134).

However, like canonical WH domain proteins, Drosophila Pcl is dependent on the α3 helix for DNA binding, with Arg631Ala, Gln634Ala and Lys637Ala mutations along this helix reducing the affinity of Pcl for DNA (34). Lys650Ala and Lys651Ala mutations (equivalent to Lys322 and Lys323 in human PHF1) in W1 of Drosophila Pcl also impair DNA binding. In human PHF1, mutation of the equivalent positions in the α3 helix and W1 (Arg304, Ser307, Asn310, Lys323 and Lys324) to glutamate, reduced the residence time on chromatin and the HMTase activity of the PRC2-PHF1 complex to a level comparable with that of the PRC2 core (34). Altogether, while both Choi *et al.* (34) and Li *et al.* (31) support a model where the WH domain of PHF1 binds to DNA via W1, a consensus has not been reached on the role of the α3 helix of the PCL proteins.

The DNA sequences preferentially bound by PCL proteins is an active area of research. While some studies report selectivity for non-methylated CpG dinucleotides (31,32), which could explain PRC2 recruitment to CpG islands, CpG targeting is insufficient to distinguish PcG target from non-target CpG islands. At this time there are no DNA motifs, other than the CpG dinucleotide, known to selectively bind PCL proteins across multiple independent studies (Table 2). However, assay variability does complicate the comparison of these studies. Li *et al.* (31) showed that the Tudor-PHD1-PHD2-EH constructs of human PHF1 and MTF2 depend on an unmethylated CpG dinucleotide for DNA binding (31). The bases immediately flanking the CpG had a small effect on affinity although a simultaneous preceding and following A/T prevented binding (Table 2, rows 28–36 for PHF1 and 39–47 for MTF2) (31). Another study confirmed the CpG dinucleotide as a determinant of binding in mammals, but they found 6–7 bp sequences were needed to accurately predict MTF2 targets from non-targets (123). Pull-down experiments using various DNA probes as a bait, complemented with ChIP-seq, supported a model where MTF2 recognises DNA shape, rather than the mere sequence motif. MTF2 target sites were predicted to have a wider minor grove and decreased propeller and helical twists, compared to methylated CpG sequences, or unmethylated but non-target CpG sequences (123). CpG dinucleotides are not required for DNA binding by the Drosophila Pcl (Table 2, rows 61 and 69). In fact, there is very little CpG methylation in Drosophila (135) without clear functional consequences (136). This may highlight the evolution of selective binding by mammalian PCL proteins to replace the role of Pho and transcription factors which contribute to the recruitment of PRC2 in Drosophila.

The reproducible trend across all studies is that PRC2-PCL complexes have greatly enhanced affinity to DNA relative to the PCL proteins alone (Table 2, compare rows 23–27 and 28–36 for PHF1 and rows 37–38 and 39–47 for MTF2) (31,32,34). Indirectly, this suggests additional contacts beyond the PCL domains which could possibly modulate the DNA-sequence selectivity. Such additional interactions could be contributed from additional regions in the PCL proteins, PRC2 core subunits or the oligomerization of PRC2. Indeed, pull-down experiments using biotinylated DNA and several MTF2 truncations demonstrated robust DNA binding requires both the WH domain and the lysine-rich region immediately C terminal to it (123). A dimeric state of PRC2 has previously been reported by one of us (137) and more recently, it was shown that PCL proteins promote the dimerization of PRC2, and this is required for high-affinity binding to DNA from the *LHX6* gene CpG island (32). This DNA was bound with higher affinity than an AT-only sequence (Table 2, compare rows 37 and 48). An independent study also showed a reduced affinity to AT-only probes, compared the LHX6 CpG probe, when using 24 and 36 bp probes (Table 2, compare rows 49 and 52, 53 and 54), but not with 48 bp sequences (Table 2, compare rows 50 and 51) (33). These experiments used a relatively long DNA with several CpG dinucleotides as well as G and C nucleotides external to CpG-dinucleotide sequences, making motif identification impossible. However, they reaffirm a role for the C and G bases in the targeting of the PRC2-PCL holo-complexes.

While PCL proteins are localised to CpG islands in vivo (31,122,123), at this time no consensus DNA sequence is sufficient to explain this localisation. Considering all the biophysical and structural data, it appears that PCL proteins bind DNA either in a sequence-independent manner (34), or with a preference for unmethylated CpG dinucleotides (31) and, at least in the case of MTF2, the shape of the DNA contributes (123). It is possible that some variation in selectivity could be explained by differences between PCL isoforms or species (31,34), additional binding sites on other PRC2 subunits like PALI1 (86), and potentially some contribution of the dimerization of PRC2 (32,137).

#### PALI1 binds to DNA and is mutually exclusive with EPOP

EPOP (C17orf96) and PALI1 (previously annotated as C10orf12) are two recently discovered members of PRC2.1 complexes (16,138,139). Their binding to PRC2 is mutually exclusive (138). EPOP overlaps with PRC2 in mouse ESCs but does not appear to enhance the recruitment of PRC2 since the depletion of EPOP leads to increased levels of SUZ12 and H3K27me3 at PcG target genes (139,140). While direct involvement of EPOP in DNA binding has not yet been reported, EPOP could indirectly affect the DNA-binding activity of the PRC2.1 complex by competing against its mutually exclusive DNA-binding accessory subunit PALI1.

PALI1 is encoded by the LCOR locus. It enhances the HMTase activity (16,141) and DNA-binding affinity (86) of PRC2. PALI1^1058-1329^, which includes the PRC2-interacting region, causes more than a 10-fold increase in the affinity of PRC2 for both mononucleosomes and naked, size-matched DNA (Table 2, compare rows 6–7 and 76–77) (86). This increase in affinity for DNA is independent of allosteric stimulation of HMTase, which is another activity of PALI1 that involves its interactions with the regulatory subunit EED (86). PRC2-PALI^1058-1250^ binds a 46 bp sequence from the CDKN2Bp CpG island and an AT-rich sequence with similar affinities, suggesting PALI1 may bind DNA non-selectively (86). PALI2 is a closely related homologue of PALI1 transcribed from the LCORL locus and, when ectopically expressed, binds to PRC2 (141). PALI2 has not been characterised in detail and it is yet to be determined if endogenous PALI2 interacts with PRC2 and regulates its activity similarly to PALI1 (86). One difference between PALI1 to PALI2 are three AT-hook motifs that were predicted in the latter (141) and could potentially bind DNA.

## DNA BINDING BY PRC1

The six PRC1 complexes in mammals can be broadly divided into canonical PRC1 (cPRC1: PRC1.2 and PRC1.4) and non-canonical PRC1 (ncPRC1: PRC1.1, PRC1.3, PRC1.5 and PRC1.6). PRC1.2 and PRC1.4 may bind RYBP or YAF2 instead of a CBX subunit and these complexes are also referred to as non-canonical PRC1. cPRC1 complexes are recruited through binding of the chromodomain of the CBX protein to H3K27me3 and overlap extensively with PRC2 (29). These sites are typically marked by H3K27me3, H2AK119Ub and are repressed (38). RING1A/B is an E3 ubiquitin ligase that forms part of all PRC1 complexes though ncPRC1 deposits most of the H2AK119Ub (38,56,57,59). ncPRC1 complexes colocalise with cPRC1 (38,57) but also have unique target genes and are not functionally redundant (38,61). Unique targets of ncPRC1 generally have higher transcription levels and may carry active histone modifications such as H3K4me3 and H3K36me3 (38,142,143). All ncPRC1 complexes can include RYBP or its homologue YAF2, and these proteins are mutually exclusive with CBX proteins in cPRC1 (15,16). RYBP binds the PRC1 product H2AK119Ub, potentially creating a positive feedback loop (144). Several PRC1-associated proteins are reported to bind DNA (Figure 1) but their role in chromatin targeting in the context of PRC1 complexes is not well understood and this is discussed below.

### PRC1.1 component KDM2B binds non-methylated CpG-rich DNA

H2AK119Ub levels in ESCs are primarily dependent on PRC1.1 (59). Studies of the recruitment mechanisms of PRC1.1 have focused on KDM2B which binds non-methylated CpG island DNA (35,36,60) and BCOR (BCL6 corepressor) which localises to BCL6 target genes (145). KDM2B demethylates H3K4me3 and H3K36me2 through a JmjC domain (146–149). KDM2B can recruit both PRC1 and PRC2 to CpG islands (56,85). However, KDM2B binds CpG islands genome-wide (35,60), which is difficult to reconcile with the establishment of Polycomb domains at only a portion of the CpG islands.

KDM2B has been shown to bind non-methylated CpG-rich DNA in vitro through its ZF-CxxC domain (35,36,60,150,151). In vivo, KDM2B is enriched at essentially all types of CpG islands; with or without promoters and is associated with active, bivalent and silent genes (35,60). Localisation of KDM2B is independent of PRC1 and the binding profile matches the closely related KDM2A which does not interact with PcG proteins (35,143). Polycomb domains are only established at a subset of KDM2B targets but how this is controlled is not understood (60).

**Figure 5.**
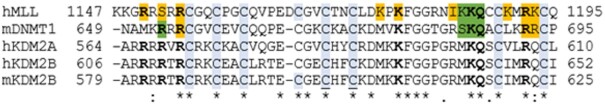
The ZF-CxxC domain of KDM2B bind CpG dinucleotides. Sequence alignment of ZF-CxxC domains. Residues highlighted in green were reported to contact the bases of the CpG dinucleotide (PDB: 2KKF (152), PDB: 3PT6 (153)). Residues in gold were reported to contact the DNA backbone. DNA-contacting residues which are conserved in human KDM2B are in bold. Conserved cysteine residues which coordinate zinc ions are highlighted in blue and two of these cysteines which mutating them disrupt KDM2B function in mice (36) are underlined.

KDM2B was first presumed to bind non-methylated CpG dinucleotides based on homology to other proteins which contain a ZF-CxxC domain. Structural studies ZF-CxxC containing proteins, Mixed Lineage Leukemia (MLL) and DNA methyltransferase 1 (DNMT1), show these domains wrap around the DNA double helix and contact both the major and minor grooves on opposite sides of the DNA (152,153). Residues that contact the DNA bases of the CpG dinucleotide are conserved between MLL to KDM2B, except for KDM2B residue Met642 (using the human KDM2B numbering, Figure 5 in green). Knockout of *KDM2B*, deletion of the ZF-CxxC domain or mutation of zinc coordinating residues in the ZF-CxxC domain (Figure 5, underlined) impairs chromatin binding by PRC1.1, causes loss of H2AK119Ub or a loss of repression at a subset of target genes (35,36,56,60,151). The ZF-CxxC domain from KDM2B preferably binds to unmethylated CpG DNA compared to methylated CpG DNA (154). More recently, it has been shown that a single CpG dinucleotide is necessary and sufficient for KDM2B DNA binding (Table 2, compare rows 80–85) (155).

KDM2A does not bind to nucleosomal DNA but rather requires a linker DNA that contains unmethylated CpG dinucleotides (156). Although it has not been directly shown, KDM2B might also bind only to non-nucleosomal DNA, which may restrict its genome occupancy. This may be because ZF-CxxC domains are reported to wrap around naked DNA (152,153) and this could be sterically prevented by a nucleosome (156).

KDM2B tethering to chromatin leads to the recruitment of PRC1.1, H2AK119Ub deposition, and subsequently the recruitment of PRC2 and its H3K27me3 mark (56,85). The recruitment of PRC2 may be mediated by JARID2 binding to H2AK119Ub, since KDM2B does not interact directly with PRC2 (16,26). PRC2 recruitment is independent of the lysine demethylase activity of KDM2B since a catalytically inactive KDM2B mutant still had this effect (85).

A recent study discovered that a long isoform of KDM2B, termed KDM2BFL, is expressed early on development, during peri-implantation (157). The short isoform is predominantly expressed post-implantation. Both the short and the long isoforms of KDM2B include the ZF-CXXC CpG-binding domains, while the demethylase domain is only present in the long isoform (157). KDM2BFL is required for the recruitment of PRCs, but this is done indirectly. During peri-implantation, KDM2BFL lead to the removal of the active H3K36me2 mark and recruits the BAF complex that opens chromatin (157). Only then, PRCs are recruited to establish H3K27me3 and H2AK119ub *de novo*. The demethylase activity of KDM2BFL is required for the establishment of PcG domains (157). Hence, the long isoform of KDM2B prepares CpG islands for the recruitment of PRCs in peri-implantation, but it does not operate as a simple tether and its mere DNA-binding activity is insufficient for that. It is tempting to speculate that later in development the short isoform of KDM2B function in keeping these CpG islands poised for the recruitment of PRC1.1. In such a model, direct interactions between PRC1.1 to the short isoform of KDM2B might improve targeting efficiency, where the subset of targeted CpG islands could be defined by additional determinants. While KDM2B and PRC1.1 localisation in cells has been studied extensively, biochemical characterisation of DNA binding of the PRC1.1-KDM2B holo-complex is lacking. As appears to be the case for holo-PRC2 complexes, it is possible that other subunits of PRC1.1 may refine targeting.

### PRC1.2/4 may bind DNA through CBX proteins

The PRC1.2 and PRC1.4 complexes are close homologs and bind the PRC2 enzymatic product H3K27me3 through a CBX protein (29). Additional recruitment mechanisms might be used by cPRC1, since some PCGF2 is retained on chromatin upon H3K27me3 depletion, but these are poorly understood (158). A heterodimer of the RING domains of RING1B and PCGF4 binds DNA with the affinity strongly dependent on DNA length, suggesting this interaction may be non-specific (Table 2, compare rows 86–95) (159). However, this may not be a cPRC1-specific function and DNA-binding activity may be conserved among the Drosophila orthologue PSC and other mammalian PCGF proteins (Table 2, rows 78–79) (160,161). CBX2 has been reported to bind DNA with the chromodomain, AT-Hook motif and serine-rich region contributing to affinity (Table 2) (162). The chromodomain of the CBX2 homologue, CBX8, also binds DNA (163). In both cases, the sequence selectivity of DNA binding was not characterised.

### PRC1.3/5 binds distinct sites to cPRC1

PRC1.3/5 complexes rarely occupy PcG target genes and instead are localised to expressed genes where they may contribute to the active state (38,164). A notable exception is the Xist RNA-mediated silencing of the inactive X chromosome (165). Relative to other PCGF proteins, a small fraction of PCGF3 is stably bound to chromatin (158). PRC1.3/5 has also been reported to deposit low levels of H2AK119Ub throughout the genome and this may be facilitated by its dynamic interactions with chromatin (59).

PRC1.3/5 interact with DNA-binding proteins but the significance of this for localisation and recruitment is not known. PCGF3 interacts with the USF1 DNA-binding transcription factor, with the depletion of USF1 and its homologue USF2 leading to the displacement of PRC1.3 (38). PCGF3 is also reported to bind DCAF7 which may link PRC1.3/5 to the zinc finger containing transcription factors ZNF503 and ZNF703 (16). The PRC1.3/5 component AUTS2 interacts with the transcription factor NRF1. NRF1 mediates the recruitment of AUTS2 to some neurodevelopmental genes during mouse motor neuron differentiation (166).

Of the PRC1 complexes, the least is known about targeting of PRC1.3/5. Due to its limited overlap with H3K27me3 and H2AK119Ub, it is unlikely it makes a significant contribution to the establishment of Polycomb domains, at least in the context of frequently studied biological systems. PRC1.3/5 may contribute to gene repression in certain cellular and biological contexts, such as X chromosome inactivation in females but more generally this appears to have divergent functions to cPRC1.

### PRC1.6 binds DNA through MAX–MGA and E2F6–DP1

PRC1.6 complexes are essential for the maintenance of pluripotency in ESCs (37,167,168). PRC1.6 overlaps with cPRC1 extensively though each complex also has unique target genes (38,169). Unique targets of PRC1.6 are not typically bound by PRC2 or marked with H3K27me3 (37,170) and include genes involved in meiosis and germ cells production (37,169,171). PCGF6 can be targeted to chromatin independently of RING1A/B, but these sites typically lack H2AK119Ub and H3K27me3 (38). In addition to PCGF6 and RING1A/B, PRC1.6 complexes comprise an E2F6–DP1 heterodimer, MAX–MGA heterodimer and L3MBTL2, which recruit PRC1.6 to unique target genes (37). The MAX–MGA and E2F6–DP1 dimers bind E-BOX (CACGTG) and E2F (GCGGGAA) DNA elements, respectively, identified through genome-wide location analysis (38,172). PCGF6 binding overlaps almost completely with MGA and tends to occur at narrow regions near the transcription start site compared to the broader distribution of RING1B and H3K27me3 (38,169,173).

The cumulative DNA-binding activities of E2F6 and MGA contribute to the targeting of PRC1.6. Deletion of the E-BOX recognising HLH domain of E2F6 or mutation of key DNA contacting residues caused a partial loss of PRC1.6 recruitment to chromatin (37,38). Likewise, knockdown of E2F6 caused loss of PRC1.6 binding at a subset of its target genes (38). The combined loss of E2F6 and the HLH domain of MGA caused a more dramatic reduction in chromatin association (38). Knockdown of MGA disrupts the association of PCGF6 with other members of the complex and causes a dramatic loss of PRC1.6 and H2AK119Ub at target genes (37,38).

L3MBTL2 is a histone binding protein also required for PRC1.6 binding to a subset of target genes (37,170,174). The Drosophila orthologue of L3MBTL2, Sfmbt, interacts with the sequence-selective DNA-binding protein Pho to recruit PRC1 to PREs (52,175,176). The region within the mammalian L3MBTL2 orthologous to the Pho binding site within the fly Sfmbt is essential for chromatin binding but does not interact with the mammalian Pho orthologue YY1 (177). L3MBTL2-dependent target genes are enriched for MGA target sequences, suggesting L3MBTL2 may play a role in MAX-MGA chromatin interaction (37).

The role of PRC1.6 in the establishment of Polycomb domains is not well understood. The sequence-specific binding of the MAX–MGA (172) and E2F6–DP1 (178) dimer may lead to H2AK119Ub deposition at repressed sites that are not bound by cPRC1 (38,169,173). However, a requirement of PRC1.6 for targeting other types of PRC1 complexes has not been shown and is likely confined to only target genes which contain MAX–MGA and E2F6–DP1 target sequences in their promotors.

### Other transcription factors proposed to recruit PRC1

PRC1 may also transiently interact with several transcription factors, but the function of these interactions is debated. CBX proteins interact with the transcription factor REST (RE1-Silencing Transcription factor) and these proteins colocalise at RE1 elements in ESCs (61,179,180). However, for binding sites less than five kilobases from a transcription start site, REST was dispensable for PRC1 recruitment (179,180). The Runx1–CBFβ complex overlap extensively with RING1B, but the knockdown of this complex affects the expression of only a small number of genes (181). The ncPRC1 subunit RYBP was previously proposed to interact with the transcription factor YY1, an observation that coined its name: Ring 1 and YY1 binding protein (182). Although mammalian YY1 has high sequence homology to the Drosophila Pho, a comparable role for YY1 in PcG protein recruitment in mammals has been excluded (54 and references therein). Despite several reports of interactions between PRC1 and transcription factors such as REST, Runx1–CBFβ and YY1, these interactions were not detected in unbiased proteomic studies that identified other PRC1 interactors (15,16). It is possible that some transcription factors contribute to the regulation of PRC1 or its target genes, either directly or indirectly. Yet, evidence in the current literature does not point towards these transcription factors as major recruiting factors of PRC1.

## DISCUSSION AND REMAINING QUESTIONS

### A proposed model for the recruitment of mammalian PcG proteins to CpG islands

PcG target promoters occur almost exclusively in non-methylated CpG islands resulting in the hypothesis that these sequences act as the elusive PREs in mammals (61,77,78). However, the mechanisms for selectively recruiting PcG proteins to these sites are incomplete and contradictory at times. DNA motifs comparable to those bound by transcription factors in Drosophila to recruit PcG proteins have not been reported. The PcG arsenal contains only two proteins where non-methylated CpG-selective DNA binding has been described in independent studies carried out by several laboratories. These are KDM2B, which forms part of the PRC1.1 complex, and the PRC2-associated PCL proteins PHF1, MTF2 and PHF19 (31,33,35,36,60,123).

**Figure 6.**
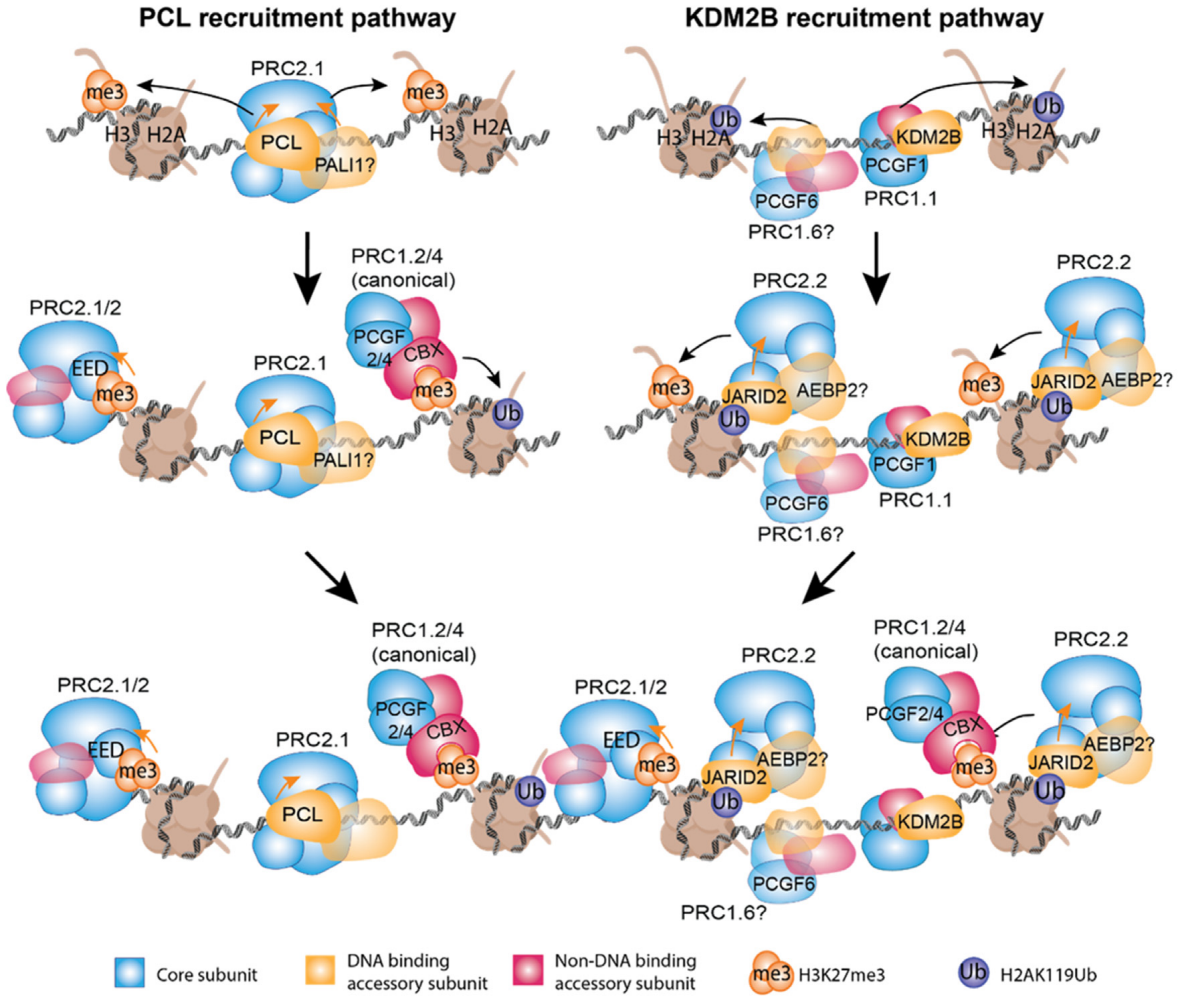
Model of DNA-dependent recruitment of PcG proteins. PRC2.1 is seeded on target DNA by the PCL proteins (top left). PRC1.1 is seeded on DNA by KDM2B (top right) and PRC1.6 may also be recruited at some sites by MGA-MAX or E2F6. Once either the H3K27me3 or the H2AK119ub marks were nucleated, they can recruit the canonical PRC1 (middle left) or the PRC2.2 (middle right), respectively. After both the H2AK119Ub and H3K27me3 repressive marks were established, positive feedback loops involving PRC1, PRC2 and their respective chromatin modifications then lead to the maintenance of the repressive marks and possibly contributed to the recruitment of other PcG proteins (bottom). Orange arrows indicate enhancement of enzymatic activity by accessory subunits (in yellow) or the H3K27me3 mark (in orange). Transparent shapes and question marks indicate uncertainty regarding the precise role taken by the indicated protein in the presented pathway. For simplicity, histone tails are only shown for one copy of H3 and H2A for each nucleosome.

Is it plausible that PcG domains at CpG islands could be established exclusively by KDM2B and PCLs? Here we discuss a model where CpG recognition by PCL-containing PRC2.1 and KDM2B-containing PRC1.1 seeds these complexes on chromatin. Positive feedback loops lead to the recruitment of other PRC1 and PRC2 complexes (Figure 6). This is far from a complete model and the remaining questions are discussed in the next sections. Most notably, this model is unable to explain the recognition of target from non-target CpG islands.

In the top left panel of Figure 6, PRC2-PCL complexes bind DNA at CpG dinucleotides and deposit H3K27me3 on nearby nucleosomes. PALI1 reportedly occurs in some PRC2.1 complexes (16). The PRC2-binding region of PALI1 increases the affinity of PRC2 for DNA but binds DNA non-selectively, at least amongst the two sequences tested (86) so may not contribute to localisation. However, PALI1 might facilitate nucleating the H3K27me3 mark by triggering an allosteric activation of PRC2.1 (86).

Once the repressive mark of PRC2 has been nucleated, histone tails carrying the H3K27me3 mark are bound by the aromatic cage of EED (Figure 6, middle left panel), causing allosteric stimulation of PRC2 and facilitating spreading of the H3K27me3 domains (27,28). EED is a core component that is found in all PRC2 complexes (Figure 1). Therefore, nucleated H3K27me3 could contribute to the recruitment or activity of additional PRC2.1 and also PRC2.2 complexes. However, despite stimulating HMTase activity, H3K27me3 has little effect on the affinity of PRC2-AEBP2 for chromatin (83) and H3K27me3 is insufficient to recruit PRC2 lacking the accessory subunit-binding modules from SUZ12 (80). Therefore, assigning a substantial direct role for H3K27me3 in the recruitment of PRC2 to CpG islands might be premature at this time. Conversely, cPRC1 is recruited by binding of H3K27me3 to the chromodomain of the CBX proteins (29,50). Some H2AK119Ub may be deposited by cPRC1 at this stage although most H2AK119Ub is attributed to ncPRC1 (38,56,57,59).

The H2AK119Ub mark can be bound by JARID2, which may lead to PRC2.2 recruitment (Figure 6, bottom panel) (26,183). JARID2 allosterically stimulates the catalytic activity of PRC2 (184) and enhances its affinity for DNA and nucleosomes (102,103,114). AEBP2 is likely also recruited at this stage since it participates in the interactions between JARID2 and a ubiquitylated nucleosome within the context of a PRC2-AEBP2-JARID2 complex (82). Furthermore, AEBP2 is required for the stable incorporation of JARID2 into PRC2 complexes (20). PRC2.2 may reinforce the deposition of H3K27me3 by PRC2.1.

In parallel, PRC1.1 is recruited to non-methylated CpG islands by KDM2B and deposits H2AK119Ub (Figure 6, top right panel) (35,36,59,60). PRC1.6 complexes include several DNA-binding proteins (16,169) and may also contribute to a DNA sequence-dependent establishment of PcG domains though this is not well understood. As described in the previous paragraph, H2AK119Ub may promote the recruitment of PRC2.2 to some PRC1 bound sites.

Having the PRC2-AEBP2-JARID2 placed downstream of ncPRC1, while cPRC1 is located downstream of PRC2, leads to the cooperation between PRC1 to PRC2 at CpG islands (Figure 6). H3K27me3 and H2AK119Ub provide convergence points for the PRC1.1, PRC2.1 and possibly PRC1.6 initiated pathways on chromatin to introduce more of the H2AK119Ub and H3K27me3 marks at CpG islands. From current evidence, it seems most DNA-binding PcG proteins either bind non-selectively (EZH2 (81,82,84) and PALI1 (86)) or recognise a low-complexity DNA motif (PCLs (31,123), AEBP2 (83,96,112), JARID2 (103,114) and KDM2B (35,60)). It is plausible to hypothesize that multiple low-complexity DNA motifs that are accessible within the context of a given nucleosome spacing or chromatin structure could simultaneously bind different PcG proteins. This view qualitatively fits with the previously proposed model for the recruitment of PRC2, where the sum of relatively weak simultaneous interactions between multiple subunits to chromatin may be required to establish PcG domains (30). Such metastable interactions could be poised on the edge of self-maintaining positive feedback loops, which could later be reinforced upon the presence of the right set of molecular cues. Hence, during cell differentiation, changes in the expression level of lineage-specific transcription factors could tip the balance between repression to derepression or vice versa. This process could take place selectively in certain loci, based on available transcription factor binding sites, rather than be driven by direct and specific interactions between PRCs to DNA. This view is also in accord with the way PREs operate in Drosophila, where they are dynamically regulated in a cell-type-specific manner (185,186). While the localisation of PcG proteins to CpG islands is clear (55,61,72–74), there are many unanswered questions on the role of DNA sequences in the recruitment of PRCs to these sites.

### Open questions on the recruitment of PcG proteins to CpG islands

#### Are there DNA target motifs of PRC1.1-KDM2B and PRC2-PCL?

The model in Figure 6 fits the available data on PcG recruitment but it fails to answer the fundamental question of how do PcG proteins distinguish target from non-target sites? There is biophysical evidence of selective CpG-binding through the ZF-CxxC domain of KDM2B (35) and the WH domain of PCL proteins (31). However, CpG dinucleotides are not a unique feature of PcG target genes, they occur throughout the genome. This is reflected in the considerable binding of KDM2B outside of PcG target genes (35,60). There is little evidence that the CpG dinucleotide is the principal determinant of binding to KDM2B and PCL within the context of PRC1 and PRC2, respectively. This is because most of the binding assays and high-resolution structures that concluded CpG-binding specificity by these proteins were carried out using truncated proteins externally to the context of their respective PRCs (Table 1).

For KDM2B, despite its genome-wide binding, PRC1 core components RING1A/B and PCGF1 are only observed at a subset of targets (35). This may be because the PRC1.1 core is recruited independently to only a subset of KDM2B targets, or the pre-formed PRC1.1-KDM2B complex may be targeted to a more limited set of genes. Quantitative DNA-binding experiments with PRC1.1, PRC1.1-KDM2B and possibly other accessory subunits (Figure 1) may hint to what extent the specific binding observed in vivo relies on specific interactions with DNA.

Unlike KDM2B, PCL proteins are predominantly detected at PcG target genes (19,187). This cannot be explained solely by the recognition of a mere CpG as reported for the WH domains (31). It has been reported that 6–7 bp including a CpG are needed to explain MTF2 localisation, and these regions were defined by DNA shape features (123). However, DNA shape could be affected by the binding of other proteins, in a mechanism referred to as DNA allostery (188). In principle, DNA allostery could modulate the binding specificity of a given factor depending on other transcription factors that are expressed in the cell and bind nearby. In vitro, the DNA-shape selectivity of MTF2 has not been investigated quantitatively and it is not known if this also applies to PHF1 and PHF19. Moreover, many of the probes that were used in previous studies (Table 2) include DNA sequences of low complexity, which could impair their hybridisation into a double-strand DNA (189). Even if perfect double-stranded DNA probes are formed, the GC content could affect their general biophysical properties, as their ability to undergo transitions from B-form to A-form (190) or to Z-form (191). Studies of DNA-binding specificity of PRC2-PCL holo-complexes are lacking despite the known DNA-binding functions of EZH1/2, EED and PALI1 that participate in these complexes (81,82,84,86,90,92). Future work in this area may identify DNA sequence motifs beyond the CpG or perhaps other chromatin-binding determinants to better explain the complex targeting of PRCs in vivo.

#### What is the role of DNA binding by PcG proteins other than KDM2B and PCLs?

In addition to the CpG binding domains of KDM2B and PCL proteins, many other PcG proteins are reported to bind DNA (Figure 1). In most cases, the DNA-binding selectivity is either disputed, such as for AEBP2 and JARID2, or has been minimally characterised, as is the case for most PRC1 complexes (see Table 1 for references).

The model proposed in Figure 6 depends on the positive feedback loops between PRC1 and PRC2 complexes to establish PcG domains. It is possible that low levels of PRC1.1-KDM2B and PRC2-PCL may be continually scanning all CpG sites throughout the genome, even if this cannot be reliably detected by methods such as ChIP-seq. The establishment of a PcG domain may also require the recruitment of PRC2.2 and cPRC1 by H2AK119Ub and H3K27me3. This might occur only at the subset of CpG sites where these complexes can also make favourable interactions with DNA and nucleosomes. At this stage, there is no reported mechanism for other PcG proteins to reinforce the binding of PRC1.1, since it is not known to interact with H3K27me3 or H2AK119Ub, although BCOR might play a role in target-specificity (192).

Some PcG domains could also require PRC1.6 for high levels of H2AK119Ub and the subsequent recruitment of other PcG proteins. PRC1.6 interacts with the MAX–MGA and E2F6–DP1 dimers which bind DNA specifically (38,172). It is unknown how these proteins contribute to the DNA-binding specificity of PRC1.6 or to the role of PRC1.6 in recruiting other PcG proteins.

In summary, it is plausible that PcG domains are dependent on the summation of DNA and chromatin recognition from many PcG proteins. Interestingly, this parallels PcG recruitment in Drosophila, where PREs are often a cluster of several transcription factor binding sites (49). If this is the case, PcG targeting in vivo would be better understood if the DNA recognition motifs and chromatin interacting domains of all the PRC1 and PRC2 holo-complexes were identified and characterised. Furthermore, the polycomb repressive deubiquitinase complexes, which remove the H2AK119Ub mark, may also play a role (193). Recent advancements in the purification of PcG holo-complexes is making it possible to address many of these questions and this is an exciting area for future studies.

### Challenges with a DNA-centric recruitment model

#### How is lineage-specific gene silencing achieved from static DNA sequences?

Histone modifications and epigenetic marks change dramatically through development while the underlying DNA sequence is fixed. Many PcG target genes are bivalent in ESCs, meaning they carry both the H3K27me3 and H3K4me3 marks, and this resolves to a monovalent state in a lineage-specific manner during differentiation (61,75). How redistribution of PcG marks is achieved is a subject of ongoing studies.

Changes in PcG protein levels may contribute to the redistribution of H3K27me3 and H2AK119Ub. PRC2 levels are high in ESCs and reduced upon differentiation along a neuronal lineage (194). This led to reduced PRC2 binding at target genes but H3K27me3 and cPRC1 were generally retained at these sites. In contrast, a greater proportion of the genome was marked by H3K27me3 in foetal lung fibroblasts than ESCs (195). This resulted from the expansion of H3K27me3 domains, that are narrower in ESCs, rather than recruitment of PRC2 to new sites. KDM2B and JARID2 are controlled by the Oct4, Nanog and Sox2 pluripotency regulators (36,97,98,196–200) and are highly expressed in embryonic stem cells but are downregulated during differentiation (36). MTF2 is the most abundant PCL protein in ESCs while PHF1 and PHF19 have higher expression levels in other tissues (131,194,201).

Some PRC1 and PRC2 sub-stochiometric members undergo alternative splicing to produce isoforms that are differentially expressed through development (36,96,141,202,203). The significance of the short isoforms and how they affect chromatin targeting is largely unknown, and in some cases not all isoforms are assembled into PRCs (203).

Overexpression of some lineage-specific PcG proteins in ESCs causes lineage-specific silencing. CBX4 and PCGF4 are enriched in neural progenitor cells relative to ESCs (194). Overexpression of these proteins in ESCs causes RING1B binding and silencing at genes normally only repressed in neural progenitor cells (194). This provides a proof of concept that changes in expression of PcG proteins can affect gene repression and highlights the value of studying lineage-specific targets in parallel with the differential expression of PRC subunits.

Cell-type specificity may be defined by epigenetic regulators outside of the PcG family. As an example, the Utf1 (Undifferentiated Embryonic Cell Transcription Factor 1) gene is expressed in ESCs but is marked with H3K27me3 during neuronal differentiation (72). The expressed state of Utf1 in ESCs is maintained by OCT4 and SOX2 binding to a nearby enhancer which prevents PRC2 recruitment. Upon differentiation, the downregulation of OCT4 and SOX2 enables the accumulation of PRC2 and consequently H3K27me3 at this site. It is unclear if this is a feature of a small number of PcG target genes or a general mechanism of modulating PcG binding, but it warrants further investigation into the way sequence-specific factors may indirectly influence PcG targeting.

Although the changes in H3K27me3, H2AK119Ub and gene expression through development have been reported for many different lineages, the mechanisms for the redistribution of these repressive marks remains poorly understood. The DNA-binding activity of PcG proteins likely defines the set of possible PcG target genes. However, the DNA-binding specificity alone cannot explain the cell type-specific distribution of PcG proteins. Target sites are likely defined and restricted by additional factors, including the expression level and activity of PcG proteins (14,194,204,205), transcription factors (72), insulators (206,207), remodelling factors (14,204,205), chromatin accessibility and nucleosome occupancy (83,92,208,209), transcriptional state (209), and overall nuclear organisation (reviewed in^210^) and possibly the local structure of chromatin, to name a few.

#### How are active promotors at CpG islands avoided by PcG proteins?

Most CpG island promoters are associated with highly expressed housekeeping genes (61,75). How PcG-mediated repression is avoided at these sites is incompletely understood. Indeed, KDM2B binding occurs at all non-methylated CpG islands, including those in the promoters of active genes (35,60). However other PcG proteins and the H3K27me3 and H2AK119Ub marks show a more restricted distribution. Several predictive models of PcG target CpG islands have been reported (61,123,211) and although these accurately identify a majority of targets in some cell contexts, none can completely explain the complexity of PcG targeting through development.

Inhibition of PRC2 by active histone marks may partially explain this. H3K27 methylation is inhibited on histone tails marked by H3K4me3 or H3K36me3 which are both features of transcribed genes (107,212). Yet, the negative effect that H3K4me3 or H3K36me3 is restricted to catalysis (107), possibly through a poor presentation of the H3 tail to the active site (82,84), with minimal effect on the affinity of PRC2 to nucleosomes (83). Hence, while active histone marks restrain the catalytic activity of PRC2, they do not fully explain how PRC2 avoids binding to CpG islands associated with active genes. It is possible that active marks antagonise to the recruitment of PRCs indirectly, given the reliance of PRCs on positive feedback loops that are dependent on their enzymatic activities. It is also plausible that other factors restrict PRC2 from active CpG islands, including an antagonism with chromatin remodelling factors (14,204,205) and RNA-mediated eviction (see below).

Large sets of CpG islands can possibly avoid polycomb-mediated gene repression given their specific location within the nucleus. Many polycomb target genes tend to cluster in certain nuclear territories or hubs that are often referred to as polycomb bodies (reviewed in Zheng and Xie (213)). This 3D structural organisation leads to long range interactions within the nucleus, and the H3K27me3 mark of PRC2 is necessary but insufficient to establish them (214). Several polycomb group proteins where implicated in the formation of large networks of intermolecular interactions and condensates. These including the PRC1 subunits PHCs (215) that can oligomerise through their SAM domain (216) and CBX2 that can form condensates (217–219). Live cell imaging in mouse embryonic stem cells led to the estimation that polycomb bodies include about ten molecules of PRC1 at a local concentration of approximately 130 nM (158). Although this concentration and number of molecules are not as high as reported in some phase separation experiments in vitro (217,219), it does demonstrate that multiple PRCs function simultaneously in a given polycomb body. Hence, the 3D organization of the genome and cooperativity between PRCs could restrain the subset of CpG islands that are targeted by polycomb-group proteins in a given cell. This model would allow a limited number of PRCs to function across the genome, consistent with their localisation being highly dynamic according to live cell imaging (158,220).

Transcription is critical in preventing PRC2, and likely therefore cPRC1, from being associated with active genes. PRC2 is dispensable for the initiation of transcriptional silencing during differentiation of ESCs but is recruited after downregulation occurs and is essential for the maintenance of the repressed state (209,221). Global transcription inhibition in ESCs leads to the recruitment of PRC2 to thousands of new genes which are normally only marked by H3K27me3 in differentiated cells (209). RNA is proposed to evict PRC2 from active genes (222–224), and several key observations support this model. First, PRC2 binds to RNA promiscuously (222), and cannot bind nucleosomes and RNA simultaneously (224). Second, PRC2 is enzymatically inhibited by RNA (124,223,225,226). Third, PRC2 is present at the promoters of genes with low expression even in cases where H3K27me3 is not deposited (222,226) and these interactions are dependent on RNA (227). While the exact mechanism for RNA-mediated regulation of PRC2 has yet to be resolved, these lines of evidence fit with a model where active transcription and possibly RNA provide the means to limit the occupancy of PRC2 on chromatin.

## CONCLUSIONS

While there is no doubt that the DNA-binding activity of PRCs is required for their recruitment to polycomb-target genes, the search for the mammalian equivalent to PREs is ongoing. It seems unlikely that mammalian PcG proteins are commonly recruited to PcG target genes by direct interactions with transcription factors that bind a well-defined DNA motif. It is also not reasonable to assume that direct interactions of high-affinity and high-specificity predominantly drive PRCs to their targets. This is because firmly docking PRCs into static DNA sequence elements would defeat their purpose as dynamic chromatin modifiers that operate across various lineages. Instead, general properties of CpG islands, such as GC richness or CpG density (61), a lack of activating signals (72,107,212) and CpG methylation status (31,35,112) combined with nucleosome positioning (83,92,209) contribute to the recruitment of PRC1 and PRC2 and promote conditions favouring the deposition of H2AK119Ub and H3K27me3 there. The PCL proteins and KDM2B provide a potential link between CpG islands and PcG proteins since they bind the CpG-dinucleotide sequences (31–36,59,60,123). However, a motif as simple as a CpG cannot explain the complexity of PcG targeting. How holo-PRCs interact with DNA and chromatin, in the context of all their subunits, remains a key question in the quest for their targeting specificity. Understanding the way multiple PcG complexes work together and potentially cooperate in the context of chromatin may allow the PRE-equivalent within mammalian CpG islands to be defined.
